# Simulated Leaching of Foliar Applied Copper Bactericides on the Soil Microbiome Utilizing Various Beta Diversity Resemblance Measurements

**DOI:** 10.1128/spectrum.01481-21

**Published:** 2022-05-10

**Authors:** A. Strayer-Scherer, S. Timilsina, Y. Y. Liao, M. Young, E. N. Rosskopf, G. E. Vallad, E. M. Goss, S. Santra, J. B. Jones, J. C. Hong, M. L. Paret

**Affiliations:** a Department of Plant Pathology, University of Florida, Gainesville, Florida, USA; b Department of Entomology and Plant Pathology, Auburn University, Auburn, Alabama, USA; c NanoScience Technology Center and Burnett School of Biomedical Science, University of Central Florida, Orlando, Florida, USA; d USDA ARS, United States Horticultural Research Laboratory, Fort Pierce, Florida, USA; e Department of Plant Pathology, Gulf Coast Research and Education Center, University of Florida, Wimauma, Florida, USA; f Department of Plant Pathology and Emerging Pathogens Institute, University of Florida, Gainesville, Florida, USA; g NanoScience Technology Center, Department of Chemistry, Materials Science and Engineering and Burnett School of Biomedical Sciences, University of Central Florida, Orlando, Florida, USA; h Department of Plant Pathology, North Florida Research and Education Center, University of Florida, Quincy, Florida, USA; University of Minnesota

**Keywords:** diversity measurements, nanomaterials, bacteria, high-throughput sequencing analysis, soil microbiology

## Abstract

Copper bactericides are routinely used to control Xanthomonas perforans (XP), causal agent of bacterial spot of tomato. Given the widespread tolerance to copper in XP strains in FL, USA, nanotechnology-based elemental composites have gained interest for their potential applications in agriculture in part due to their enhanced antimicrobial properties and toxicity to copper-tolerant strains. However, little is known about the potential impact of conventional copper bactericides as well as nano-based elemental composites on soil microbial communities, as determined by high-throughput sequencing of the 16S rDNA. We compared the effects of 2 and 200 μg/mL of core-shell (CS), a metallic copper composite, and a conventional copper bactericide + mancozeb (Cu+Man) on the soil microbiome. These treatments were compared to three controls, the microbial profile of the soil prior to application of copper products, a water application, and spiking the soil with a soilborne phytobacterium, Ralstonia solanacearum (RS). The RS treatment was included to determine if downstream analysis could detect the artificial inoculation. Utilizing multiple β diversity measurements, each emphasizing various tenets of ecology, provided a greater perspective of the effects the treatments had on the microbiome. Analysis of HTS data revealed that the two treatments containing field applied rates of metallic copper, CS 200 and Cu+Man, had the largest impact on the soil microbiome at seven-days posttreatment compared to water. However, we simulated field applied rates of CS 200 entering the soil by treating soil with CS 2 and determined this concentration had a negligible effect on the soil microbiome.

**IMPORTANCE** Nanotechnology-based elemental composites have gained popularity for their potential applications in plant disease management due to their enhanced antimicrobial properties. However, little is known about their potential impact on the environment. Foliar applications of nano metallic composites upon leaching into the soil have the potential to impact soil microbial populations that in turn influence soil health. Utilizing multiple β diversity measurements, high-throughput sequencing analysis revealed that field applied rates of metallic copper (200 μg/mL) from an advanced copper composite (core-shell [CS]) and a conventional copper bactericide in combination with mancozeb had the largest impact on the soil microbiome compared to water and nontreated control. To simulate leaching from the leaf surface, a lower concentration (2 μg/mL) of CS was also applied to the soil and had a negligible effect on the soil microbiome. Thus, field applied rates of CS may have a minimal effect on soil microbial communities.

## INTRODUCTION

Copper-based compounds have been used to manage plant pathogens for centuries, due to high biocidal properties (1–3). Several different formulations of copper have been developed to manage a number of plant-pathogenic bacteria, fungi, and oomycetes (3). Commercialized copper bactericides in general contain insoluble, micron-sized metallic copper compounds (3–5). However, due to continuous use, several plant-pathogenic bacterial species have developed resistance to copper, which reduces the efficacy of copper-based bactericides (6–8). Nanotechnology-based, elemental composites are gaining popularity for their potential applications in agriculture, including for enhanced antimicrobial activity of conventional elements used as bactericides, such as copper.

Nanometer-sized metallic compounds have higher antimicrobial activity compared to their micron-sized counterparts (9–12). This increase in antibacterial activity is likely due to the small size and high surface-to-volume ratio of metallic nanoparticles (11, 13, 14). Due to their heightened antibacterial activity, several studies have evaluated the use of nanomaterials to manage copper resistant *Xanthomonas* species that cause bacterial spot of tomato (15–19). In a previous study, three copper composites (core-shell copper, CS; multivalent copper, MV; and fixed quaternary ammonium copper, FQ) designed utilizing nanomaterials and were evaluated as potential alternatives to commercial copper bactericides for controlling bacterial spot caused by copper-tolerant strains of *X. perforans* (18). For each of these composites, copper particles are released in a slow manner and embedded in a hydrophilic, porous silica gel matrix to reduce particle-particle aggregation, improve overall dispersion, bioavailability, and antimicrobial activity of copper ions in the composites (20, 21). *In vitro* assays revealed that 100 μg/mL of metallic copper from CS and FQ completely inhibited bacterial growth of a copper-tolerant *X*. *perforans* strain within 1 h of exposure, whereas MV inhibited bacterial growth within 24 h of exposure. In contrast, none of the micron-sized metallic copper rates (100–1000 μg/mL) from Kocide 3000 significantly reduced copper-tolerant *X*. *perforans* populations compared to the water control after 48 h of exposure (18). In greenhouse experiments, 100, 500, and 1000 μg/mL rates of metallic copper from CS significantly reduced bacterial spot disease severity compared to the grower standard (copper at 540 μg/mL + manganese/zinc ethylene bis-dithiocarbamate [Cu+Man]). In contrast, only the 500 and 1000 μg/mL rates of metallic copper from FQ and MV were able to significantly reduce disease severity compared to the grower standard in all three greenhouse experiments (18). In field studies, foliar applications of the copper composites significantly reduced disease severity compared to water controls and used 80% less metallic copper in comparison to the grower standard (18). In a separate study, foliar applications of FQ were as effective at controlling bacterial and fungal citrus diseases such as citrus canker (*X. citri* subsp. *citri*), scab (*Elsionë fawcettii*), and melanose (*Diaporthe citri*) as conventional copper-based compounds (22). These studies highlighted the potential use of copper composites created using materials in the nano-size as a promising alternative to micron-size copper-based bactericides to manage plant diseases.

The use of nanomaterials for managing plant pathogens is a rapidly expanding area of research, but little is known about their potential impact on the environment; particularly their effects on soil microbial communities and potential toxicity to the ecosystem (23). The effects of nanomaterials on the soil microbiome are of major interest because the diversity of microbial populations in soil can influence soil health and crop productivity (24–27). The difference between populations among sites is defined as β diversity, which is related to α diversity, the biodiversity within individual sites, and γ diversity the aggregate of the community within the study (28). Thus, calculating β diversity is apropos for observing the effect of stimuli on the microbiome. Resemblance measurements quantify the association or similarity between units by a function which assigns this association a real number (29), calculating β diversity. However, different methods for measuring β diversity have been described and each measurement has distinctive properties that emphasize various aspects of the sampled populations (30, 31). Anderson et al., (2010) underscored that multiple resemblance measures can yield different results, as these different methods correspond to differing ecological hypotheses for calculating β diversity. Thus, measuring β diversity phylogenetically, by using UniFrac (32) or by relative abundance or total abundance, by using Bray-Curtis (33) or Euclidean (30), respectively, or to include joint absences by using Gower (34) or Euclidean, will provide greater insights into the community and the effect a stimulus has on a community. This study provides a unique opportunity to compare various methods for calculating β diversity by comparing the effects of the copper products on the bacterial community, observing temporal changes, and to determine if these analyses can detect the infestation of soil with a bacterium that is known to survive in this ecological niche.

Since copper compounds have broad-spectrum antimicrobial activity against pathogenic and nonpathogenic bacteria (1–3, 35), runoff foliar applications of copper nanoparticles may significantly impact microbial populations in the soil compared to their micron-sized counterparts (23). Thus, the goal of this study was to compare the effects an advanced copper composite, CS (18) to micron-sized metallic copper (Cu) in combination with manganese/zinc ethylene bis-dithiocarbamate (Man), the growers’ standard, on the composition of the soil microbiome. In tomato and pepper production, Man is applied in combination with copper bactericides to improve efficacy against copper-tolerant strains of *Xanthomonas* (7, 36). Due to its high antibacterial activity *in vitro* and efficacy against bacterial spot of tomato under greenhouse and field conditions, CS was selected for use in this study (18). The objectives of this study were to characterize the soil bacterial composition following treatment with copper composites to each other and temporally by using i) HTS to provide an in-depth characterization of the bacterial populations which were impacted by the treatments, and ii) multiple resemblance measurements that represent differing ecological hypothesis to determine which treatments significantly impacted the bacterial community.

## RESULTS

### The effect of copper bactericides on taxonomic composition of soil bacterial communities observed by sequencing.

A total of 24,141,022 OTUs was generated in this study of which 11,620,125 and 12,520,897 OTUs were generated in trials 1 and 2, respectively. HTS results were first analyzed to determine the diversity within samples. A SIMPROF test determined that in trial 1 Pi = 2.737 (*P*-value of 0.001) and in trial 2 Pi = 3.467 (*P* value of 0.001). Based on Spearman rank correlation of the 0 DPT dry soil data sets, the two trials had a correlation of 0.114 and an R-value of 1 (*P*-value = 0.029) calculated by ANOSIM, indicating that the beginning populations of the collected soil for the two trials were very different from each other, most likely due to collection of the soil for trial 1 in the spring and for trial 2 in the winter (Fig. S1, S2). Thus, the trial data sets were analyzed separately. The variance in diversity for each treatment was measured at each time point as previously stated. The Shannon diversity values among treatments were not significantly different in either of the two trials (Table 1; Fig. S1).

**TABLE 1:** The effect of copper composite (core-shell silica copper, CS); copper-mancozeb (Cu+Man), and Ralstonia solanacearum (RS) at 5 × 10^8^ CFU/mL, and water (H_2_O) compared to the nontreated control (NT/No H_2_O at 0 DPT) on the soil microbial composition in the first and second growth chamber experiments (trial 1 and trial 2, respectively)^*a*^

	Shannon diversity trial 1		Shannon diversity trial 2	
Treatment, Cu concn	1 DPT^*b*^	7 DPT^*b*^	1 DPT^*b*^	7 DPT^*b*^
CS, 2 μg/mL	2.66 a	2.80 a	2.55 a	2.56 a
CS, 200 μg/mL	2.69 a	2.71 a	2.63 a	2.70 a
Cu+Man, 540 μg/mL	2.47 a	2.66 a	2.65 a	2.56 a
RS	2.46 a	2.50 a	2.47 a	2.45 a
H_2_O	2.59 a	2.46 a	2.53 a	2.62 a
NT/No H_2_O at 0 DPT	2.49 a	2.49 a	2.49 a	2.49 a

a Samples were collected at 1 and 7 days post treatment (DPT).

b Column means indicated with the same letters are not significantly different (*P* < 0.05) based on SNK (Student Newman-Keuls) statistical analysis in the IBM SPSS package.

**FIG 1:**
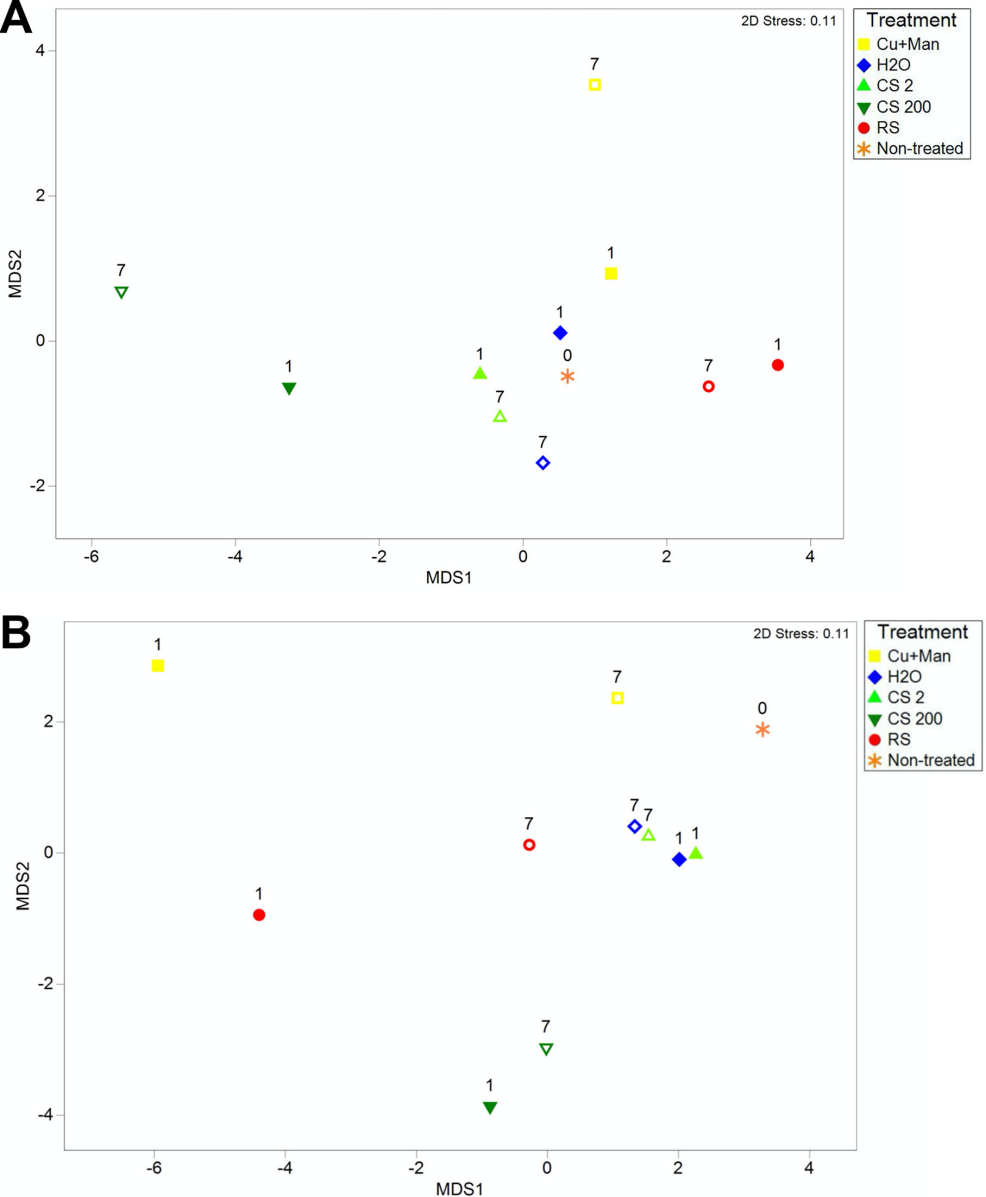
Metric multidimensional scaling plots (mMDS) with an unrestricted zero intercept comparing the distance among the centroids of bacterial population, observed by sequencing 16s rDNA, after being treated with either metallic copper at 2 and 200 μg/mL from core-shell silica copper (CS 2 (light-green triangle) and CS 200 (dark-green triangle), respectively), copper-mancozeb (yellow square), Ralstonia solanacearum (RS; red circle), or with water (H_2_O; blue diamond) and a nontreated control (No H_2_O at 0 DPT [orange star]). Soil samples were taken at 0 (orange star), 1 (open shape), or 7 (filled in shape) days post treatment (DPT). Data sets were transformed to the 4th root and analyzed by Bray Curtis. Trial 1 (A) and trial 2 (B) were conducted April and December 2016, respectively.

**FIG 2:**
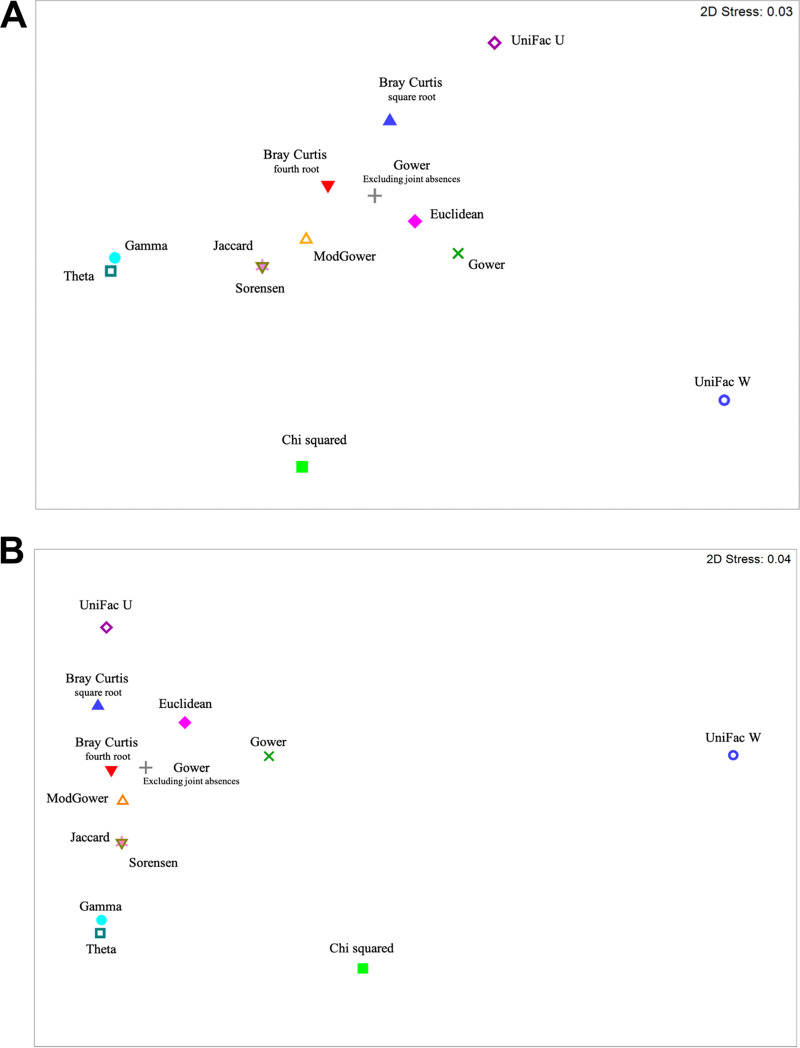
Nonmetric multidimensional scaling plot displaying the differences between different resemblance measures to calculate the β diversity derived from 2STAGE analysis, which compressed the high-throughput sequencing results from the soil bacterial communities treated with metallic copper at 2 and 200 μg/mL from core-shell silica copper, copper-mancozeb, Ralstonia solanacearum, with water and nontreated controls from the first (A) and second (B) growth chamber experiments.

β diversity was visualized in mMDS plots for each measurement (Fig. 1 and Fig. S3). Compared to the nontreated control at 0 DPT, Cu + Man and CS 200 caused the greatest shift in bacterial populations in the soil in both trials (Fig. 1). In trial 1, bacterial populations in Cu + Man and CS 200 treated soil continued to shift away from the nontreated control at 7 DPT (Fig. 1A). In contrast, the bacterial populations became more similar to the nontreated control at 7 DPT for both the Cu + Man and CS 200 (Fig. 1B). In each trial, CS 2 and sterile water had a similar effect on the soil microbiome compared to the nontreated control (Fig. 1), which was later confirmed statistically. The spiked control (RS) also had an effect on the soil microbiome at 1 DPT, but the populations became more similar to the nontreated control at 7 DPT in both trials (Fig. 1). To compare the different methods for calculating β diversity, 2STAGE analysis using Spearman rank correlation was conducted for each trial. This showed that UniFrac W, a phylogenetic measurement, was the least similar to the other resemblance matrices (Fig. 2; Tables S1 and S2). Multivariate measurements that emphasize relative or log abundances grouped together, which included Bray Curtis 4th root, ModGower, Jaccard, and Sorensen. Bray Curtis square root and Euclidean were more distantly related. Gower (00), which excludes joint absences was more similar to Bray Curtis 4th root, compared to Gower which was more similar to Euclidean. The other distance measurement, Chi squared, was the second furthest from the Bray Curtis 4th root. Theta, Gamma, and UniFrac U, phylogenetic measurements, were distantly related.

PERMANOVA was used to statistically analyze the β diversity data set and compared ‘treatments’, ‘days’, and the interaction between the two factors (Table S3). For both trials, neither UniFrac U nor W were significant for any of the factors for α = 0.05. In trial 1, ‘treatment’ was significant for the remaining resemblance measures. Likewise, for trial 2, ‘treatment’ was significant for the rest of the resemblance measures except for Gamma (*P* = 0.106). For trial 2, ‘day’ was significant for Bray Curtis transformed at the 4th root, Euclidean, Theta, Gower, Gower (00), ModGower, Sorensen, and Jaccard.

Pairwise comparison was performed to determine which treatments and sampling dates were significantly different, as the PERMANOVA results indicated that the microbiome was significantly altered by the soil treatments and for the temporal samples depending on the β diversity analysis. Focus was given to the comparisons between soil treatment versus the water control at 1 and 7 DPT, soil treatment versus the nontreated control, soil treatment 1 DPT versus soil treatment 7 DPT, and then comparison of the different dilutions of CS at the different temporal sampling (Table S4). Considering that the microbiomes of trial 1 and 2 differed, particular attention was given to the pairwise comparisons that were significant for both trials with α = 0.1 (Table 2). Of all the methods for determining the β diversity, the resemblance measures Bray Curtis transformed to the 4th root, Euclidean, Gamma, Chi squared, Gower, and Gower (00) indicated that the bacterial communities were altered for both trials. CS 200 and Cu+Man were significantly different from the water control when samples were taken at 7 DPT, data transformed at 4th root and analyzed with Bray Curtis and Euclidean. In general, the effects of CS 200 and Cu+Man may not be immediate, but a gradual response. Additionally, both CS 200 and Cu+Man were significantly different from CS 2 and from each other at 7 DPT. Euclidean and Chi squared, which emphasize rare taxa, detected that RS was significantly different than water 1 DPT. Treatments CS 200 and Cu+Man were significantly different from water and CS 2 at 7 DPT when analyzed by Euclidean. CS 200 at 1 and 7 DPT were different from the water control and CS 2 at 7 DPT for Chi squared. Gamma, which focuses on phylogenetic differences, showed that CS 200 was significantly different from water 1 DPT. Both Gower and Gower (00) indicated that Cu+Man was different from water at 7 DPT, and that CS 2 was different from CS 200 at 7 DPT. Additionally, Cu+Man was different from CS 2 at 7 DPT for Gower.

**TABLE 2:** Values of pairwise *t* test based on a variety of resemblance measures analyzing 16s rDNA sequences by high-throughput sequencing comparing soil treatments and their effects on the soil microbiome where both trial 1 and trail 2 were significantly different α = 0.1

Multivariate measure^*a*^	D1 trt versus H_2_O^*b*^				D7 trt versus H_2_O^*b*^				D1 trt versus trt^*b*^				D7 trt versus trt^*b*^			
	Trial	Trt	t	P	Trial	Trt	t	P	Trial	Trt	t	P	Trial	Trt	t	P
Bray Curtis	NS				1	CS 200	1.44	0.0291	NS				1	CS 2 versus CS 200	1.36	0.0288
					2	CS 200	1.20	0.0891					2	CS 2 versus CS 200	1.29	0.0289
					1	Cu+Man	1.28	0.0305					1	CS 2 versus Cu+Man	1.26	0.0265
					2	Cu+Man	1.15	0.0596					2	CS 2 versus Cu+Man	1.28	0.0280
													1	CS 200 versus Cu+Man	1.53	0.0265
													2	CS 200 versus Cu+Man	1.36	0.0275
Euclidean	1	RS	1.21	0.0555	1	CS 200	1.27	0.0298	NS				1	CS 2 versus CS 200	1.23	0.0325
	2	RS	1.31	0.0284	2	CS 200	1.19	0.0300					2	CS 2 versus CS 200	1.21	0.0281
	NS				1	Cu+Man	1.20	0.0283					1	CS 2 versus Cu+Man	1.21	0.0303
					2	Cu+Man	1.01	0.0284					2	CS 2 versus Cu+Man	1.16	0.0285
Gamma	1	CS 200	1.40	0.0299	NS				NS				NS			
	2	CS 200	1.36	0.0537												
Chi squared	1	CS 200	1.06	0.0297	1	CS 200	1.16	0.0281	NS				1	CS 2 versus CS 200	1.12	0.0262
	2	CS 200	1.05	0.0290	2	CS 200	1.07	0.0587					2	CS 2 versus CS 200	1.08	0.0266
	1	RS	1.06	0.0291												
	2	RS	1.06	0.0291												
Gower excluding joint absences	NS				1	Cu+Man	1.22	0.0299	NS				1	CS 2 versus CS 200	1.32	0.0303
					2	Cu+Man	1.09	0.0601					2	CS 2 versus CS 200	1.20	0.0301
Gower	NS				1	Cu+Man	1.22	0.0303	NS				1	CS 2 versus CS 200	1.21	0.0292
					2	Cu+Man	1.00	0.0860					2	CS 2 versus CS 200	1.24	0.0317
													1	CS 2 versus Cu+Man	1.15	0.0833
													2	CS 2 versus Cu+Man	1.21	0.0337

a Data were transformed to the 4th root.

b Treatments (Trt) consisted of soil treated with metallic copper at 2 and 200 μg/mL from core-shell silica copper (CS 2 and CS 200, respectively), copper-mancozeb (Cu+Man), Ralstonia solanacearum (RS), with water (H_2_O) the first trial (1) and second (2) growth chamber experiments. NS = Indicating that the comparisons were not significant for neither trial 1 nor trial 2.

## DISCUSSION

Due to their high-biocidal properties, copper-based bactericides are commonly used in agriculture to manage plant diseases in a wide variety of crops (3, 35, 37). However, extensive applications of copper have led to increased risk of copper toxicity, soil accumulation, leaching into water tables and aquatic ecosystems, and resistant bacterial pathogens (6–8, 38). Due to risks to nontarget organisms, the EPA has recommended reducing the single maximum application rates of 11 different copper compounds such as copper hydroxide in cucurbit, onion, pepper, walnut, and apple production (39). Although tomato is not currently included on this list, it could be added in the future, as tomato growers in Florida applied approximately between 27,500 and 61,000 kg of metallic copper in 2019 (10 to 22 applications per acre per season; 26,000 harvested acres; 30% of Kocide 3000 is metallic copper). Bactericides containing nanometer-sized metallic copper could be a promising alternative to their commercial micron-sized copper-based bactericides, due their increased antimicrobial activity at lower concentrations (18, 19, 22). In a previous field study, foliar applications of three advanced copper composites (CS, MV, and FQ) significantly reduced bacterial spot disease severity compared to water controls using 80% less metallic copper in comparison to the grower standard (Cu+Man) (18). If these advanced copper composites are labeled for use in the near future, the average amount of metallic copper applied to fresh-market tomatoes could be reduced to approximately 9,000 kg/year in Florida. Thus, potentially reducing the amount of copper accumulating in the soil and leaching into water tables. However, the use of nanomaterials for managing plant pathogens is a rapidly expanding area of research and little is known about their potential impact on the environment; particularly their effects on the soil microbial communities that can influence soil health and crop productivity (23–27).

In this study, we compared for the first time the temporal effect on the soil microbiome following exposure to micron-or nano-sized copper-based bactericides. Possibly due to field soil being collected at two different times of the year, the bacterial populations were drastically different prior to and following treatment with the copper compounds. This was likely due to seasonal changes effecting the soil microbiome (40, 41). The differing populations for both trials provided the opportunity to observe the effect of CS, Cu+Man, and RS on diverse communities. Both CS 200 and Cu+Man had a relatively longer lasting effects on the microbiome. It is interesting that for trial 1 the populations of C2 200 and Cu+Man at 7 DPT were more diverse than at 1 DPT, while for trial 2 the 7 DPT samples became more similar to the controls, indicating that the soil treatments might vary depending on the original bacterial population or seasonality could have an effect. It should also be noted that when CS is applied at 200 μg/mL, spatial movement and dilution caused by rain events, watering, and soil moisture could quickly dilute CS 200 to CS 2. This in turn would have a negligible effect on the microbiome and would be similar to adding water, as CS 2 and the water control were very similar. On the other hand, the mobility of these materials in the soil and their potential for accumulation is unknown. Thus, additional experiments with extended temporal and spatial sampling, adding a hundredfold dilution of Cu+Man, and comparing growth chamber results to field trials would be informative.

Each resemblance measurement emphasizes a different aspect for measuring β diversity (31), UniFrac compares the communities phylogenetically (32) while Bray-Curtis (33) or Euclidean (34) compares the relative or total abundance, respectively. Using multiple β diversity methods provided a greater insight into the effect of treatments on the soil bacterial community over time as each resemblance measurement emphasizes different properties of the compared communities. For example, CS 200 compared to water 1 DPT were significantly different for both Gamma, a phylogenetic based Bray-Curtis presence/absence resemblance measure (42), and Chi squared which emphasizes rare species (43) (Table 2). This could indicate that phylogenetically diverse rare taxa changed between the two samples and would provide an indication to which types of taxa should be the targeted. Joint absences, where shared missing taxa is considered informative, are ideal for studies where the hypothesis is to determine disappearance or consequences of invasions (30). Both Gower and Euclidean (34), resemblance measures that consider joint absences, showed that Cu+Man was significantly different from the water control at 7 DPT. These two resemblance measures and Bray Curtis 4th root were the only resemblance measures that showed significant differences between Cu+Man and CS 2 at 7 DPT. Interestingly, this was not observed with Gower (00) which could indicate that Cu+Man reduced some taxa below the detectable limit compared to soil treated with water or CS 2. Both Gower and Euclidean also showed a significant difference between CS 2 versus CS 200 at 7 DPT, which indicated taxa disappeared when the soil was treated with greater concentrations of CS.

Interestingly, only Euclidean and Chi squared measurements determined that RS 1 DPT was significantly different from water 1 DPT (Table 2). Raw data transformed on log(y + 1) or to the 4th root and analyzed with Euclidean identify changes in total abundance; the total number of taxa (30). Compared to Bray Curtis which calculates the relative abundances, the evenness of distribution of individuals among species in a community, and if one of the compared samples has a significant loss of taxa, Bray Curtis becomes unstable (44). Thus, when Chi squared is paired with Euclidean, the observed changed in the community were due to rare taxa and a change in the total number of taxa. Further studies are needed to replicate this finding to determine if utilization of these two resemblance measurements can detect the introduction of a population into an environment from HTS data sets. If it holds true, then these methods for determining β diversity could assist in many aspects of microbial ecology, including, but not limited to pathogen screening, environmental impact reclamation, or detection of bacterial contamination.

## MATERIALS AND METHODS

### Bacterial strains and storage.

Ralstonia solanacearum strain RS5 was used as a spike control to test the sensitivity of the β diversity analyses. R. solanacearum is a soilborne bacterial plant pathogen that can cause disease in over 200 plants worldwide (45). This strain was isolated from tomato in Florida and characterized as biovar 1, phylotype II (45). For short-term storage and experiments, bacteria were grown on nutrient agar (NA) medium (BBL, Becton, Dickinson and Co., Cockeysville, MD, USA) at 28°C for 24 h. For long-term storage, purified cultures were stored in a sterile 30% glycerol solution at −80°C.

### Design of the copper composites.

The copper composite, CS, was designed to reduce particle-particle aggregation, improve overall dispersion, bioavailability, and antimicrobial activity of copper ions, as previously described (18). Briefly, copper particles are imbedded in a hydrophilic, porous silica gel matrix to reduce particle-particle aggregation during the sol-gel synthesis process (4). Copper was chelated by the silica gel and partially converted to crystalline copper hydroxide/oxide particles at pH 7.5 and above. Colloidal silica particles (46) were used as a filler to further disperse copper-silica gel particles in the CS composite (47).

### Growth chamber experimental design.

10 liters of soil were collected at a depth of 0–15 cm from four separate locations in a tomato production field at the University of Florida, North Florida Research and Education Center in Quincy, FL, in April 2016 and December 2016 for first and second growth chamber experiments, respectively. The soil was mixed to create a bulk soil sample (total volume 40 liters) and incubated at 28°C with 12 h light and dark periods for 24 h. After 24 h, the bulk soil sample was divided and transferred to 10-cm diameter pots (400 mL of soil/pot) with sterile, polystyrene 100 mm x 15 mm petri plates (Fisherbrand, Thermo Fisher Scientific Inc, Waltham, MA, USA) at the bottom of the pots to prevent runoff. All 10-cm diameter pots (eight pots per treatment) were placed into clear plastic storage bins and the lids were closed at night to maintain soil moisture. To prevent the soil from drying, potted soil was sprayed every morning with a fine mist of sterile tap water using a conventional hand sprayer. After 48 h at 28°C and immediately prior to treatment application, 10 g of soil were destructively sampled from four randomly selected pots (nontreated control) and stored in 50 mL centrifuge tubes (Thermo Fisher Scientific Inc, Waltham, MA, USA) at −80°C until further analysis. The following treatments were prepared in 400 mL of sterile tap water for the growth chamber experiment, which was replicated: metallic copper prepared from CS at rates of 2 and 200 μg/mL (CS 2 and CS 200, respectively); Kocide 3000 (2.1 g/L) and Penncozeb 75DF (United Phosphorus, Inc., King of Prussia, PA, USA; 1.2 g/L) (Cu+Man); RS5 bacterial suspension (5 × 10^8^ CFU/mL; [RS; spiked control]); and sterile tap water (water control). In a completely randomized design, 50 mL of each treatment were poured into eight pots to saturate the soil. At 1 and 7 days post treatment (DPT), 10 g of soil were removed from each pot (four pots per treatment per sample time) and stored in 50 mL centrifuge tubes at −80°C until further analysis.

### Soil DNA extraction.

Two DNA extractions were performed on each soil sample using the PowerSoil DNA isolation kit (Mo Bio Laboratories, Inc., Carlsbad, CA, USA). The DNA extraction protocol was modified as follows to include an added phenol chloroform step to improve DNA quality as previously described (48). The DNA concentration and quality for each sample was determined using a Nanodrop 2000 Spectrophotometer (Thermo Fisher Scientific Inc, Waltham, MA, USA). Extracted DNA was stored at −20°C until further analysis.

### Illumina MiSeq library generation and 16S sequencing.

Four DNA samples from the nontreated control at 0 DPT and each of the treatments at 1 and 7 DPT were selected for 16S rRNA sequencing. The DNA concentration for each sample was confirmed using a Qubit 3 Fluorometer (Thermo Fisher Scientific Inc, Waltham, MA, USA). Illumina MiSeq metagenomic libraries were prepared following the protocol as previously described and using forward primer V2 [5′-ACTGGCGGACGGGTGAGTAA-3′] and reverse primer V3 [5′- CGTATTACCGCGGCTGCTGG-3′] that were tagged and barcoded (49, 50). Prepared libraries were sequenced in Illumina MiSeq with 2 × 300 bp read length at Interdisciplinary Center for Biotechnology Research, University of Florida.

### Sequence analysis.

The paired raw sequence reads (*.fastq format) were curated as previously described (51, 52) using MOTHUR pipeline v.1.38.1 (53). The fastq files were quality filtered, screened, and denoised. Sequences shorter than 250 bp or with more than eight homopolymers were removed from the library. Sequences containing ambiguous bases were also removed. Chimeric sequences were removed using UCHIME (54). The taxonomy of sequences was determined using Bayesian classifier (55) against SILVA 16S sequence database (56). Sequences were clustered into OTUs using nearest-neighbor joining algorithm at 97% identity and the taxonomy was assigned to the clustered sequences.

### Statistical analysis.

Data were analyzed using the PRIMER software package v7.0 (42). The resulting OTUs were transformed to square root or the fourth square root. The HTS data sets from both trials were tested with similarity profile analysis (SIMPROF) to determine if the observed similarities were significant or due to chance (57). SIMPROF was conducted on a Bray-Curtis similarity matrix with 9999 permutations and plot limits set at 99%. Alpha diversity was determined by calculating the Shannon diversity index using natural log transformation. Resemblance matrices were created using Bray-Curtis similarity, Euclidean, Chi squared, Gamma, Theta, Gower, Gower excluding joint absences (Gower [00]), ModGower, Sorensen, and Jaccard (42). ModGower was performed using log_10_ transformation, UniFrac weighted (UniFrac W) and unweighted (UniFrac U) were performed as described by Schloss et al. (2009) and Lozupone and Knight (2005) (53, 58). Heatmaps were created by averaging the four repeated treatments per day for each trial. For this analysis, only phyla that were identified and were present in at least two or more samples were included. Cluster analysis was performed on a resemblance matrices created by Bray-Curtis or Euclidean analysis. The samples were compared to each other by running SIMPROF with significance set at 0.05, and 9,999 permutations. Metric multidimensional scaling plots (mMDS) compared the distance among the centroids and an unrestricted zero intercept. Permutational multivariate analysis of variance (PERMANOVA), and PERMANOVA pairwise comparison were the statistical approaches used to determine if the diversity observed between groups was greater or equal to the diversity within groups (Tables S1-S2) (42, 59). PERMANOVA was conducted with a main test of 9999 permutations, and permutations of residuals under a reduced model. 2STAGE set with Spearman rank for correlation (60) was used to compare the two trials and to compare the relationship of the resemblance measures. In order to compare all the resemblance measurements, UniFrac W results were converted to similarity by (1-ω_i_). Similarity of the two trials was calculated by Spearman rank correlation and Analysis of Similarities (ANOSIM) (42).

### Data availability.

16S rRNA sequencing that supports the findings of this study have been deposited in the NCBI database under BioProject accession number PRJNA801413.
