# Supplementary material for: Simulated Leaching of Foliar Applied Copper Bactericides on the Soil Microbiome Utilizing Various Beta Diversity Resemblance Measurements
Source: Microbiol Spectr. 2022 May 10;10(3):e01481-21. doi: 10.1128/spectrum.01481-21 (PMC9241806; doi:10.1128/spectrum.01481-21)
Supplement: SUPPLEMENTAL FILE 1 — Supplemental material. Download spectrum.01481-21-s001.pdf, PDF file, 1.7 MB [file spectrum.01481-21-s001.pdf]

Table S1. Spearman rank for correlation for resemblance measurements analyzing the effect of different concentrations of core-shell silica copper nanomaterials, copper-mancozeb, water, and inoculated with *Ralstonia solanacearum* sampled at one- and seven-days post treatment compared to non-treated soil taken at day zero on soil bacterial communities characterized by rDNA 16s for trial 1.

	Bray Curtis square root	Bray Curtis 4th root	Chi Squared	Euclidean	Gamma	Gower excluding joint absences	Gower	Jaccard	ModGower	Sorensen	Theta	UniFrac U	UniFrac W
Bray Curtis square root	1.0												
Bray Curtis 4th root	0.907	1.0											
Chi Squared	0.271	0.410	1.0										
Euclidean	0.810	0.839	0.417	1.0									
Gamma	0.344	0.521	0.414	0.386	1.0								
Gower excluding joint absences	0.859	0.915	0.463	0.912	0.450	1.0							
Gower	0.679	0.708	0.477	0.901	0.324	0.901	1.0						
Jaccard	0.550	0.824	0.509	0.629	0.622	0.728	0.552	1.0					
ModGower	0.723	0.935	0.495	0.781	0.596	0.869	0.687	0.961	1.0				
Sorensen	0.550	0.824	0.509	0.629	0.622	0.728	0.552	1.0	0.961	1.0			
Theta	0.300	0.483	0.411	0.353	0.995	0.406	0.285	0.605	0.568	0.605	1.0		
UniFrac U	0.665	0.512	0.270	0.594	0.076	0.593	0.539	0.279	0.396	0.279	0.057	1.0	
UniFrac W	-0.012	-0.065	0.005	0.341	-0.165	0.221	0.483	-0.119	-0.035	-0.119	-0.175	0.014	1.0

Table S2. Spearman rank for correlation for resemblance measurements analyzing the effect of different concentrations of core-shell silica copper nanomaterials, copper-mancozeb, water, and inoculated with *Ralstonia solanacearum* sampled at one- and seven-days post treatment compared to non-treated soil taken at day zero on soil bacterial communities characterized by rDNA 16s for trial 2.

	Bray Curtis square root	Bray Curtis 4th root	Chi Squared	Euclidean	Gamma	Gower excluding joint absences	Gower	Jaccard	ModGower	Sorensen	Theta	UniFrac U	UniFrac W
Bray Curtis square root	1.0												
Bray Curtis 4th root	0.891	1.0											
Chi Squared	0.122	0.269	1.0										
Euclidean	0.791	0.815	0.240	1.0									
Gamma	0.458	0.622	0.309	0.461	1.0								
Gower excluding joint absences	0.833	0.940	0.346	0.883	0.590	1.0							
Gower	0.548	0.617	0.430	0.823	0.429	0.806	1.0						
Jaccard	0.717	0.945	0.356	0.749	0.670	0.912	0.622	1.0					
ModGower	0.596	0.870	0.421	0.651	0.685	0.834	0.570	0.977	1.0				
Sorensen	0.596	0.870	0.421	0.651	0.685	0.834	0.570	0.977	1.0	1.0			
Theta	0.422	0.593	0.321	0.438	0.995	0.560	0.410	0.649	0.671	0.671	1.0		
UniFrac U	0.737	0.657	-0.040	0.662	0.330	0.655	0.435	0.566	0.477	0.477	0.308	1.0	
UniFrac W	-0.332	-0.392	-0.122	-0.142	-0.221	-0.274	-0.001	-0.382	-0.387	-0.387	-0.218	-0.327	1.0

Table S3. PERMANOVA results of soil bacterial communities, characterized by rDNA 16s, sequencing treated with different concentrations of core-shell silica copper nanomaterials, copper-mancozeb, water, and inoculated with *Ralstonia solanacearum* sampled at one- and seven-days post treatment compared to non-treated soil taken at day zero analyzed by various multivariate measurements.

Multivariate measure [a]	Trial [b]	Source [c]	df	Sum of Square	Mean of Square	Pseudo-F	P(perm) [d]
Bray Curtis Square root	1	Tr	4	1924.9	481.21	2.3845	0.001
		Da	1	190.43	190.43	0.94359	0.3821
		TrxDa	4	687.83	171.96	0.85206	0.6566
		Residual	33	6659.8	201.81		
		Total	43	9633			
	2	Tr	4	2126.7	531.68	2.243	0.0011
		Da	1	372.23	372.23	1.5703	0.1215
		TrxDa	4	1038	259.49	1.0947	0.3232
		Residual	33	7822.3	237.04		
		Total	43	11765			
Bray Curtis	1	Tr	4	1719.7	429.92	2.0988	0.0001
		Da	1	213.46	213.46	1.0421	0.3472
		TrxDa	4	771.68	192.92	0.94181	0.6227
		Residual	33	6759.7	204.84		
		Total	43	9671.1			
	2	Tr	4	1742.6	435.65	1.769	0.0014
		Da	1	412.85	412.85	1.6764	0.0538
		TrxDa	4	1063.1	265.79	1.0792	0.2996
		Residual	33	8127.1	246.27		
		Total	43	11737			
Euclidean	1	Tr	4	756.14	189.03	1.9318	0.0001
		Da	1	101.69	101.69	1.0392	0.3466
		TrxDa	4	366.78	91.695	0.93705	0.7151
		Residual	33	3229.2	97.855		
		Total	43	4557.8			
	2	Tr	4	712.92	178.23	1.7384	0.0001
		Da	1	144.23	144.23	1.4068	0.0485
		TrxDa	4	428.93	107.23	1.0459	0.3048
		Residual	33	3383.3	102.53		
		Total	43	4827.2			
Chi Squared	1	Tr	4	4.222	1.0555	1.354	0.0001
		Da	1	0.85048	0.85048	1.091	0.2511

		TrxDa	4	3.0712	0.76781	0.98497	0.6264
		Residual	33	25.724	0.77953		
		Total	43	34.588			
	2	Tr	4	3.7069	0.92673	1.1485	0.0036
		Da	1	0.88564	0.88564	1.0976	0.2391
		TrxDa	4	3.1687	0.79218	0.98177	0.6429
		Residual	33	26.627	0.80689		
		Total	43	35.571			
Gamma	1	Tr	4	25.778	6.4444	1.8883	0.0001
		Da	1	2.9373	2.9373	0.86069	0.6235
		TrxDa	4	11.81	2.9526	0.86517	0.7863
		Residual	33	112.62	3.4127		
		Total	43	156.34			
	2	Tr	4	19.435	4.8587	1.4471	0.1064
		Da	1	5.4007	5.4007	1.6085	0.1498
		TrxDa	4	18.002	4.5006	1.3404	0.1443
		Residual	33	110.8	3.3576		
		Total	43	160.9			
Theta	1	Tr	4	25.368	6.342	1.916	0.0001
		Da	1	2.8718	2.8718	0.86758	0.6254
		TrxDa	4	11.811	2.9527	0.89203	0.752
		Residual	33	109.23	3.3101		
		Total	43	152.46			
	2	Tr	4	14.943	3.7357	1.4259	0.0459
		Da	1	4.3766	4.3766	1.6705	0.0798
		TrxDa	4	13.706	3.4265	1.3079	0.0871
		Residual	33	86.458	2.6199		
		Total	43	125.1			
UniFrac U	1	Tr	4	19660	4915.1	0.99951	0.533
		Da	1	4917.8	4917.8	1.0001	0.5445
		TrxDa	4	19672	4918.1	1.0001	0.5281
		Residual	33	1.62E+05	4917.6		
		Total	43	2.11E+05			
	2	Tr	4	19654	4913.6	0.99944	0.5282
		Da	1	4915.7	4915.7	0.99986	0.5471
		TrxDa	4	19665	4916.1	0.99995	0.5294
		Residual	33	1.62E+05	4916.4		
		Total	43	2.11E+05			
UniFrac W	1	Tr	4	0.049701	0.012425	1.0084	0.5041

		Da	1	-0.0002465	-0.0002465	Negative	
		TrxDa	4	0.14191	0.035477	2.8793	0.1135
		Residual	33	0.40661	0.012321		
		Total	43	0.63072			
	2	Tr	4	19903	4975.7	0.99751	0.5392
		Da	1	4969.5	4969.5	0.99628	0.5527
		TrxDa	4	19941	4985.3	0.99943	0.5392
		Residual	33	1.65E+05	4988.1		
		Total	43	2.14E+05			
Gower	1	Tr	4	5277.2	1319.3	1.7088	0.0001
		Da	1	812.1	812.1	1.0519	0.3358
		TrxDa	4	3002.2	750.55	0.97217	0.5514
		Residual	33	25477	772.04		
		Total	43	35368			
	2	Tr	4	1468.7	367.16	1.5886	0.0009
		Da	1	358.59	358.59	1.5515	0.0411
		TrxDa	4	941.84	235.46	1.0188	0.3956
		Residual	33	7626.9	231.12		
		Total	43	10831			
Gower excluding joint absences	1	Tr	4	1751.4	437.84	1.8357	0.0002
		Da	1	250.74	250.74	1.0513	0.3335
		TrxDa	4	906.18	226.55	0.94984	0.6131
		Residual	33	7870.8	238.51		
		Total	43	11025			
	2	Tr	4	4960	1240	1.552	0.0013
		Da	1	1136	1136	1.4219	0.0542
		TrxDa	4	3276	819.01	1.0251	0.382
		Residual	33	26365	798.95		
		Total	43	36986			
ModGower	1	Tr	4	0.54001	0.135	1.7087	0.0001
		Da	1	0.079079	0.079079	1.0009	0.4517
		TrxDa	4	0.29879	0.074696	0.94543	0.7148
		Residual	33	2.6073	0.079008		
		Total	43	3.607			
	2	Tr	4	0.52237	0.13059	1.4474	0.0012
		Da	1	0.13413	0.13413	1.4866	0.0288
		TrxDa	4	0.37248	0.093121	1.0321	0.3577
		Residual	33	2.9774	0.090224		
		Total	43	4.1312			

Jaccard	1	Tr	4	3933.5	983.38	1.6175	0.0001
		Da	1	579.25	579.25	0.95279	0.5848
		TrxDa	4	2264.1	566.03	0.93106	0.7727
		Residual	33	20062	607.95		
		Total	43	27472			
	2	Tr	4	3689.1	922.26	1.316	0.003
		Da	1	1042.3	1042.3	1.4873	0.0209
		TrxDa	4	2864	715.99	1.0217	0.3887
		Residual	33	23126	700.79		
		Total	43	31649			
Sorensen	1	Tr	4	1580	395	1.7674	0.0001
		Da	1	210.48	210.48	0.94182	0.5826
		TrxDa	4	823.75	205.94	0.92148	0.7772
		Residual	33	7375	223.49		
		Total	43	10223			
	2	Tr	4	1495	373.76	1.3496	0.0139
		Da	1	459.05	459.05	1.6576	0.0261
		TrxDa	4	1160.3	290.08	1.0474	0.3426
		Residual	33	9138.9	276.94		
		Total	43	12639			

[a.] All data was transformed at the 4th square root unless otherwise noted.

[b.] Trial 1 and trial 2 were conducted April and December 2016, respectively.

[c.] Comparisons were abbreviated as treatment = Tr and Days post application = Da

[d] Consisted of 9,999 permutation

Table S4. PERMANOVA pairwise comparison of soil bacterial populations after treatment with different concentrations of core-shell copper nanomaterials, copper-mancozeb, water, or addition of *Ralstonia solanacearum* bacterial cell suspension, at one- and seven-days post treatment, and non-treated soil taken at day zero as analyzed by various multivariate measurements.

Multivariate measure[a]	Trial[b]	Comparison[c]	Treatment[d]	t	P-value (perm)
Bray Curtis square root	1	D 1 vs H_2O D 1	CS 2	0.75496	0.6598
			CS 200	1.062	0.3764
			Cu+Man	0.75216	0.625
			RS	1.1576	0.2591
		D 7 vs H_2O D 7	CS 2	0.88593	0.4858
			CS 200	1.5211	0.0274
			Cu+Man	1.2623	0.1153
			RS	1.2218	0.1761
		D 1 vs No H_2O D 0	CS 2	0.82272	0.6592
			CS 200	1.2327	0.1758
			Cu+Man	0.84094	0.5976
			RS	1.1776	0.2519
			H_2O	0.74374	0.7419
		D 7 vs No H_2O D 0	CS 2	0.86204	0.6385
			CS 200	1.6161	0.0264
			Cu+Man	1.2353	0.1419
			RS	1.0797	0.3165
			H_2O	0.86764	0.5397
		D 1 vs D 7	CS 2	0.84209	0.5386
			CS 200	1.0841	0.2524
			Cu+Man	1.1317	0.2287
			RS	0.83322	0.8862
			H_2O	0.77155	0.683
		Trt D 1 vs Trt D 1	CS 2 vs CS 200	0.94526	0.4943
			CS 2 vs Cu+Man	0.92521	0.5046
			CS 200 vs Cu+Man	1.3613	0.1138
		Trt D 7 vs Trt D 7	CS 2 vs CS 200	1.5089	0.0291
			CS 2 vs Cu+Man	1.3585	0.0564
			CS 200 vs Cu+Man	1.7803	0.0291

	2	D 1 vs H_2O D 1	CS 2	0.76196	0.8247
			CS 200	1.634	0.0256
			Cu+Man	1.1434	0.2015
			RS	1.587	0.0868
		D 7 vs H_2O D 7	CS 2	1.0053	0.3728
			CS 200	1.2941	0.1137
			Cu+Man	1.3514	0.0596
			RS	1.0137	0.3994
		D 1 vs No H_2O D 0	CS 2	1.1502	0.2537
			CS 200	2.2545	0.0291
			Cu+Man	1.1181	0.1998
			RS	1.7782	0.0284
			H_2O	1.2283	0.1159
		D 7 vs No H_2O D 0	CS 2	1.3364	0.0539
			CS 200	1.6137	0.0278
			Cu+Man	1.5462	0.0314
			RS	1.3981	0.1168
			H_2O	1.0843	0.3761
		D 1 vs D 7	CS 2	0.93123	0.4927
			CS 200	1.0951	0.3129
			Cu+Man	1.1474	0.2001
			RS	1.007	0.3373
		Trt D 1 vs Trt D 1	CS 2 vs CS 200	1.6721	0.0272
			CS 2 vs Cu+Man	1.1382	0.1728
			CS 200 vs Cu+Man	1.1778	0.1466
		Trt D 7 vs Trt D 7	CS 2 vs CS 200	1.3665	0.0596
			CS 2 vs Cu+Man	1.4732	0.0254
			CS 200 vs Cu+Man	1.3991	0.0325
Bray Curtis	1	D 1 vs H_2O D 1	CS 2	0.90837	0.6575
			CS 200	1.1463	0.1964
			Cu+Man	0.87885	0.6902
			RS	1.173	0.1442
		D 7 vs H_2O D 7	CS 2	0.93111	0.6191

		CS 200	1.4378	0.0291
		Cu+Man	1.2757	0.0305
		RS	1.1267	0.1178
	D 1 vs No H_2O D 0	CS 2	0.89832	0.7051
		CS 200	1.2182	0.1145
		Cu+Man	1.0075	0.3506
		RS	1.2146	0.0562
		H_2O	0.91004	0.689
	D 7 vs No H_2O D 0	CS 2	0.98357	0.4002
		CS 200	1.4935	0.0291
		Cu+Man	1.2269	0.0817
		RS	1.0795	0.2255
		H_2O	0.98168	0.4478
	D 1 vs D 7	CS 2	0.89931	0.7774
		CS 200	1.0593	0.3461
		Cu+Man	1.0955	0.2088
		RS	0.92289	0.9182
		H_2O	0.89989	0.5694
	Trt D 1 vs Trt D 1	CS 2 vs CS 200	0.92875	0.6293
		CS 2 vs Cu+Man	1.0448	0.3112
		CS 200 vs Cu+Man	1.3022	0.0568
	Trt D 7 vs Trt D 7	CS 2 vs CS 200	1.3564	0.0288
		CS 2 vs Cu+Man	1.2629	0.0265
		CS 200 vs Cu+Man	1.5346	0.0265
2	D 1 vs H_2O D 1	CS 2	0.93518	0.7113
		CS 200	1.3636	0.0288
		Cu+Man	1.1157	0.1972
		RS	1.423	0.0279
	D 7 vs H_2O D 7	CS 2	0.94636	0.6611
		CS 200	1.204	0.0891
		Cu+Man	1.1525	0.0596
		RS	0.97259	0.5706
	D 1 vs No H_2O D 0	CS 2	1.1011	0.1946

			CS 200	1.7413	0.0292
			Cu+Man	1.1154	0.1989
			RS	1.6251	0.0279
			H_2O	1.1508	0.14
		D 7 vs No H_2O D 0	CS 2	1.246	0.0271
			CS 200	1.4196	0.0284
			Cu+Man	1.279	0.0313
			RS	1.2772	0.0575
			H_2O	1.0887	0.1414
		D 1 vs D 7	CS 2	1.0307	0.3143
			CS 200	1.0432	0.4008
			Cu+Man	1.1173	0.1739
			RS	1.1668	0.1337
			H_2O	0.97296	0.4816
		Trt D 1 vs Trt D 1	CS 2 vs CS 200	1.4393	0.0285
			CS 2 vs Cu+Man	1.1259	0.1711
			CS 200 vs Cu+Man	1.075	0.253
		Trt D 7 vs Trt D 7	CS 2 vs CS 200	1.2905	0.0289
			CS 2 vs Cu+Man	1.2757	0.028
			CS 200 vs Cu+Man	1.3625	0.0275
Euclidean	1	D 1 vs H_2O D 1	CS 2	0.93669	0.6354
			CS 200	1.129	0.1141
			Cu+Man	0.95636	0.5242
			RS	1.2136	0.0555
		D 7 vs H_2O D 7	CS 2	0.96432	0.5775
			CS 200	1.2727	0.0298
			Cu+Man	1.195	0.0283
			RS	1.1412	0.0516
		D 1 vs No H_2O D 0	CS 2	0.93885	0.6858
			CS 200	1.2046	0.1149
			Cu+Man	1.0077	0.3653
			RS	1.2124	0.0268
			H_2O	0.94919	0.5126

	D 7 vs No H_2O D 0	CS 2	0.99565	0.3395
		CS 200	1.3724	0.0296
		Cu+Man	1.1739	0.0848
		RS	1.1177	0.1119
		H_2O	0.98572	0.4247
	D 1 vs D 7	CS 2	0.93421	0.8072
		CS 200	1.0254	0.343
		Cu+Man	1.0869	0.1417
		RS	0.93448	0.9445
		H_2O	0.90976	0.7448
	Trt D 1 vs Trt D 1	CS 2 vs CS 200	0.97025	0.5497
		CS 2 vs Cu+Man	1.0533	0.2738
		CS 200 vs Cu+Man	1.3017	0.0302
	Trt D 7 vs Trt D 7	CS 2 vs CS 200	1.2337	0.0325
		CS 2 vs Cu+Man	1.2109	0.0303
		CS 200 vs Cu+Man	1.4233	0.0285
2	D 1 vs H_2O D 1	CS 2	0.97824	0.5991
		CS 200	1.237	0.027
		CS 2	CS 2	CS 2
		RS5	1.3174	0.0284
	D 7 vs H_2O D 7	CS 2	0.96071	0.7422
		CS 200	1.1891	0.03
		Cu+Man	1.0989	0.0284
		RS	1.0876	0.1098
	D 1 vs No H_2O D 0	CS 2	1.0751	0.1145
		CS 200	1.5117	0.028
		Cu+Man	1.0965	0.2034
		RS	1.4599	0.0304
		H_2O	1.0967	0.1464
	D 7 vs No H_2O D 0	CS 2	1.1457	0.0288
		CS 200	1.3205	0.0282
		Cu+Man	1.2026	0.0283
		RS	1.2402	0.0314
		H_2O	1.053	0.1481

		D 1 vs D 7	CS 2	1.0176	0.2894
			CS 200	1.0328	0.2863
			Cu+Man	1.0985	0.2019
			RS	1.1048	0.1759
			H_2O	1.0043	0.4281
		Trt D 1 vs Trt D 1	CS 2 vs CS 200	1.2799	0.0278
			CS 2 vs Cu+Man	1.1219	0.2015
			CS 200 vs Cu+Man	1.1252	0.1093
		Trt D 7 vs Trt D 7	CS 2 vs CS 200	1.2092	0.0281
			CS 2 vs Cu+Man	1.1648	0.0285
			CS 200 vs Cu+Man	1.2765	0.0262
Chi squared	1	D 1 vs H_2O D 1	CS 2	0.98166	0.8263
			CS 200	1.0619	0.0297
			Cu+Man	0.98691	0.656
			RS	1.0698	0.0291
		D 7 vs H_2O D 7	CS 2	0.97906	0.7157
			CS 200	1.1637	0.0281
			Cu+Man	1.0938	0.057
			RS	1.0248	0.1688
		D 1 vs No H_2O D 0	CS 2	0.97162	0.8574
			CS 200	1.0919	0.0301
			Cu+Man	1.0244	0.1761
			RS	1.1016	0.027
			H_2O	1.0089	0.4245
		D 7 vs No H_2O D 0	CS 2	0.98587	0.6584
			CS 200	1.1907	0.0274
			Cu+Man	1.0856	0.0571
			RS	1.0412	0.1439
			H_2O	1.0077	0.3706
		D 1 vs D 7	CS 2	0.9604	0.9173
			CS 200	1.0114	0.3439
			Cu+Man	1.039	0.1129
			RS	0.97303	0.9166

		H_2O	0.98946	0.6354
	Trt D 1 vs Trt D 1	CS 2 vs CS 200	0.96871	0.914
		CS 2 vs Cu+Man	1.0059	0.4035
		CS 200 vs Cu+Man	1.0926	0.0286
	Trt D 7 vs Trt D 7	CS 2 vs CS 200	1.1187	0.0262
		CS 2 vs Cu+Man	1.0398	0.1193
		CS 200 vs Cu+Man	1.1545	0.0291
2	D 1 vs H_2O D 1	CS 2	0.99922	0.4534
		CS 200	1.0562	0.029
		Cu+Man	0.98757	0.6213
		RS	1.0671	0.0291
	D 7 vs H_2O D 7	CS 2	0.98927	0.5965
		CS 200	1.0767	0.0587
		Cu+Man	1.0198	0.1427
		RS	1.0158	0.3433
	D 1 vs No H_2O D 0	CS 2	1.0225	0.2013
		CS 200	1.1512	0.0318
		Cu+Man	1.0163	0.3185
		RS	1.1356	0.0281
		H_2O	1.0365	0.1719
	D 7 vs No H_2O D 0	CS 2	1.0599	0.0301
		CS 200	1.1136	0.028
		Cu+Man	1.0331	0.1156
		RS	1.0906	0.0271
		H_2O	1.0428	0.0849
	D 1 vs D 7	CS 2	1.0182	0.2014
		CS 200	1.0047	0.4363
		Cu+Man	1.0011	0.5403
		RS	1.0633	0.0294
		H_2O	1.0065	0.4589
	Trt D 1 vs Trt D 1	CS 2 vs CS 200	1.0832	0.027
		CS 2 vs Cu+Man	1.0187	0.1704
		CS 200 vs Cu+Man	1.0128	0.3221

		Trt D 7 vs Trt D 7	CS 2 vs CS 200	1.0838	0.0266
			CS 2 vs Cu+Man	1.0596	0.089
			CS 200 vs Cu+Man	1.1348	0.0304
Gamma	1	D 1 vs H_2O D 1	CS 2	1.1899	0.1162
			CS 200	1.3988	0.0299
			Cu+Man	1.4635	0.0297
			RS	0.86938	0.8012
		D 7 vs H_2O D 7	CS 2	0.76017	0.9733
			CS 200	1.145	0.1712
			Cu+Man	0.69661	0.9709
			RS	1.0768	0.292
		D 1 vs No H_2O D 0	CS 2	1.0395	0.4519
			CS 200	1.4911	0.0312
			Cu+Man	1.3039	0.0613
			RS	0.87227	0.7349
			H_2O	0.88227	0.8009
		D 7 vs No H_2O D 0	CS 2	0.77522	0.9422
			CS 200	1.2403	0.057
			Cu+Man	1.0143	0.4102
			RS	1.1789	0.1704
			H_2O	0.91966	0.6859
		D 1 vs D 7	CS 2	0.81877	0.9704
			CS 200	0.95378	0.6227
			Cu+Man	1.0598	0.3971
			RS	0.92972	0.7434
			H_2O	0.84135	0.8057
		Trt D 1 vs Trt D 1	CS 2 vs CS 200	1.2581	0.0879
			CS 2 vs Cu+Man	1.0565	0.3158
			CS 200 vs Cu+Man	1.3142	0.0552
		Trt D 7 vs Trt D 7	CS 2 vs CS 200	0.88895	0.7153
			CS 2 vs Cu+Man	0.9537	0.5495
			CS 200 vs Cu+Man	1.1852	0.1717
	2	D 1 vs H_2O D 1	CS 2	0.93764	0.6583

			CS 200	1.3559	0.0537
			Cu+Man	1.3602	0.1449
			RS	1.2702	0.0865
		D 7 vs H_2O D 7	CS 2	0.89551	0.6012
			CS 200	1.2278	0.2185
			Cu+Man	1.0427	0.3994
			RS	1.4331	0.0839
		D 1 vs No H_2O D 0	CS 2	1.1319	0.2537
			CS 200	1.8689	0.0304
			Cu+Man	1.5015	0.0276
			RS	1.4956	0.0285
			H_2O	1.1798	0.204
		D 7 vs No H_2O D 0	CS 2	1.3247	0.1342
			CS 200	1.4968	0.0313
			Cu+Man	1.2849	0.0295
			RS	1.8671	0.0278
			H_2O	1.4926	0.0269
		D 1 vs D 7	CS 2	1.1716	0.2285
			CS 200	0.80634	0.8872
			Cu+Man	1.154	0.0567
			RS	1.4226	0.0267
			H_2O	1.1506	0.2584
		Trt D 1 vs Trt D 1	CS 2 vs CS 200	1.2758	0.2052
			CS 2 vs Cu+Man	1.2739	0.0296
			CS 200 vs Cu+Man	0.86309	0.7491
		Trt D 7 vs Trt D 7	CS 2 vs CS 200	1.1483	0.2349
			CS 2 vs Cu+Man	1.0577	0.3742
			CS 200 vs Cu+Man	1.2342	0.0859
Theta	1	D 1 vs H_2O D 1	CS 2	0.77289	0.9697
			CS 200	1.1767	0.1695
			Cu+Man	0.71185	0.9705
			RS	1.0891	0.2573
		D 7 vs H_2O D 7	CS 2	1.2032	0.0859

		CS 200	1.4253	0.0298
		Cu+Man	1.4877	0.0292
		RS	0.88212	0.7347
	D 1 vs No H_2O D 0	CS 2	0.78968	0.9446
		CS 200	1.2648	0.0314
		Cu+Man	1.0235	0.4001
		RS	1.1752	0.1751
		H_2O	0.93655	0.681
	D 7 vs No H_2O D 0	CS 2	1.048	0.4524
		CS 200	1.4984	0.0284
		Cu+Man	1.3125	0.0596
		RS	0.88029	0.6865
		H_2O	0.90324	0.7739
	D 1 vs D 7	CS 2	0.82615	0.9706
		CS 200	0.97646	0.5966
		Cu+Man	1.0743	0.394
		RS	0.91622	0.7393
		H_2O	0.86588	0.7987
	Trt D 1 vs Trt D 1	CS 2 vs CS 200	0.89444	0.7125
		CS 2 vs Cu+Man	0.963	0.5097
		CS 200 vs Cu+Man	1.2135	0.1463
	Trt D 7 vs Trt D 7	CS 2 vs CS 200	1.2565	0.1138
		CS 2 vs Cu+Man	1.0642	0.3163
		CS 200 vs Cu+Man	1.3265	0.0932
2	D 1 vs H_2O D 1	CS 2	0.95273	0.6589
		CS 200	1.2978	0.0246
		Cu+Man	1.2928	0.1423
		RS	1.1169	0.2296
	D 7 vs H_2O D 7	CS 2	0.91447	0.5774
		CS 200	1.1891	0.2331
		Cu+Man	1.0612	0.3997
		RS	1.4163	0.0849
	D 1 vs No H_2O D 0	CS 2	1.1298	0.2648

			CS 200	1.7901	0.0247
			Cu+Man	1.3884	0.0282
			RS	1.3215	0.0562
			H_2O	1.1869	0.2024
		D 7 vs No H_2O D 0	CS 2	1.3154	0.1405
			CS 200	1.407	0.0543
			Cu+Man	1.2834	0.0562
			RS	1.8052	0.0294
			H_2O	1.4997	0.0287
		D 1 vs D 7	CS 2	1.1714	0.2235
			CS 200	0.85073	0.8906
			Cu+Man	1.1301	0.1454
			RS	1.4402	0.0286
			H_2O	1.1657	0.2275
		Trt D 1 vs Trt D 1	CS 2 vs CS 200	1.2236	0.1954
			CS 2 vs Cu+Man	1.2535	0.0545
			CS 200 vs Cu+Man	0.96518	0.5697
		Trt D 7 vs Trt D 7	CS 2 vs CS 200	1.1234	0.2237
			CS 2 vs Cu+Man	1.0615	0.3817
Unifrac U	1	D 1 vs H_2O D 1	CS 2	0.96898	0.7975
			CS 200	1.1296	0.1393
			Cu+Man	0.931	0.8277
			RS	1.0971	0.151
		D 7 vs H_2O D 7	CS 2	0.99	0.4851
			CS 200	1.2876	0.0259
			Cu+Man	1.2143	0.0563
			RS	1.0441	0.3107
		D 1 vs No H_2O D 0	CS 2	0.91216	0.9133
			CS 200	1.1123	0.0846
			Cu+Man	1.084	0.1708
			RS	1.1733	0.0273
			H_2O	0.99414	0.5132
		D 7 vs No H_2O D 0	CS 2	1.0282	0.3364

		CS 200	1.2786	0.0271
		Cu+Man	1.1474	0.0545
		RS	1.0434	0.3206
		H_2O	1.0317	0.3427
	D 1 vs D 7	CS 2	0.90439	0.9404
		CS 200	0.97179	0.5883
		Cu+Man	1.0151	0.4669
		RS	0.94401	0.8256
		H_2O	0.97138	0.6102
	Trt D 1 vs Trt D 1	CS 2 vs CS 200	0.88373	0.9141
		CS 2 vs Cu+Man	1.0659	0.1167
		CS 200 vs Cu+Man	1.1552	0.0565
	Trt D 7 vs Trt D 7	CS 2 vs CS 200	1.1676	0.0573
		CS 2 vs Cu+Man	1.1261	0.0827
		CS 200 vs Cu+Man	1.2575	0.0293
2	D 1 vs H_2O D 1	CS 2	1.0002	0.0829
		CS 200	0.99972	1
		Cu+Man	0.99992	0.8325
		RS	0.99991	0.829
	D 7 vs H_2O D 7	CS 2	0.99998	0.8307
		CS 200	0.99987	0.8302
		Cu+Man	0.99979	0.9724
		RS	0.99984	0.8833
	D 1 vs No H_2O D 0	CS 2	0.99999	0.5963
		CS 200	0.99944	1
		Cu+Man	0.99995	0.7392
		RS	0.99977	0.9704
		H_2O	0.99999	0.6543
	D 7 vs No H_2O D 0	CS 2	0.99992	0.8257
		CS 200	0.99977	1
		Cu+Man	0.99985	0.9708
		RS	0.99981	0.9112
		H_2O	0.99996	0.6628

		D 1 vs D 7	CS 2	1	0.4862
			CS 200	0.99991	0.9123
			Cu+Man	0.99988	0.9708
			RS	1.0001	0.3155
			H_2O	0.99999	0.6279
		Trt D 1 vs Trt D 1	CS 2 vs CS 200	0.99965	1
			CS 2 vs Cu+Man	0.99996	0.8072
			CS 200 vs Cu+Man	0.99975	1
		Trt D 7 vs Trt D 7	CS 2 vs CS 200	0.99995	0.8557
			CS 2 vs Cu+Man	0.9998	0.9735
			CS 200 vs Cu+Man	0.9998	1
Unifrac W	1	D 1 vs H_2O D 1	CS 2	1.0001	0.5183
			CS 200	1	0.5953
			Cu+Man	1.0001	0.3432
			RS	1	0.6039
		D 7 vs H_2O D 7	CS 2	0.99997	0.7145
			CS 200	0.99964	0.9696
			Cu+Man	0.99989	0.8224
			RS	0.99997	0.739
		D 1 vs No H_2O D 0	CS 2	1	0.5992
			CS 200	1	0.6056
			Cu+Man	1.0001	0.257
			RS	1	0.5143
			H_2O	1.0001	0.2229
		D 7 vs No H_2O D 0	CS 2	1.0001	0.458
			CS 200	0.99967	0.9454
			Cu+Man	0.99991	0.7484
			RS	0.99998	0.6312
			H_2O	1.0001	0.4866
		D 1 vs D 7	CS 2	1.0001	0.4514
			CS 200	0.99999	0.6524
			Cu+Man	0.99999	0.5978
			RS	1.0001	0.2359

		H_2O	1.0001	0.5751
	Trt D 1 vs Trt D 1	CS 2 vs CS 200	1.0001	0.5082
		CS 2 vs Cu+Man	1	0.5231
		CS 200 vs Cu+Man	0.99988	0.7407
	Trt D 7 vs Trt D 7	CS 2 vs CS 200	0.99989	0.8311
		CS 2 vs Cu+Man	0.9999	0.7672
		CS 200 vs Cu+Man	0.99965	1
2	D 1 vs H_2O D 1	CS 2	1.0012	0.2552
		CS 200	0.99579	1
		Cu+Man	1.0041	0.1189
		RS	1.0012	0.2339
	D 7 vs H_2O D 7	CS 2	0.99983	0.8033
		CS 200	1.0006	0.3977
		Cu+Man	0.99627	1
		RS	0.9999	0.6226
	D 1 vs No H_2O D 0	CS 2	0.99986	1
		CS 200	0.99307	1
		Cu+Man	1.0044	0.029
		RS	1.0022	0.1149
		H_2O	1.0019	0.085
	D 7 vs No H_2O D 0	CS 2	0.99997	1
		CS 200	0.99962	0.5385
		Cu+Man	0.99886	0.7387
		RS	0.99971	0.7068
		H_2O	0.9997	0.9388
	D 1 vs D 7	CS 2	0.99993	1
		CS 200	0.99877	0.8823
		Cu+Man	1.0004	0.3938
		RS	0.99793	0.9154
		H_2O	1.0005	0.4007
	Trt D 1 vs Trt D 1	CS 2 vs CS 200	0.99397	1
		CS 2 vs Cu+Man	1.0026	0.0867
		CS 200 vs Cu+Man	0.99851	0.8535

		Trt D 7 vs Trt D 7	CS 2 vs CS 200	1.002	0.1147
			CS 2 vs Cu+Man	0.99803	0.9698
			CS 200 vs Cu+Man	0.99796	1
Gower	1	D 1 vs H_2O D 1	CS 2	0.93814	0.6317
			CS 200	1.0986	0.2305
			Cu+Man	0.89719	0.6854
			RS	1.089	0.2591
		D 7 vs H_2O D 7	CS 2	0.95261	0.5756
			CS 200	1.3724	0.0291
			Cu+Man	1.2203	0.0299
			RS	1.0687	0.1999
		D 1 vs No H_2O D 0	CS 2	0.93113	0.6854
			CS 200	1.1434	0.1712
			Cu+Man	1.0241	0.3213
			RS	1.1421	0.0865
			H_2O	0.94172	0.5647
		D 7 vs No H_2O D 0	CS 2	0.9931	0.3707
			CS 200	1.3968	0.0303
			Cu+Man	1.192	0.0536
			RS	1.0639	0.2853
			H_2O	0.98464	0.4303
		D 1 vs D 7	CS 2	0.93709	0.7436
			CS 200	1.064	0.3121
			Cu+Man	1.0643	0.2291
			RS	0.95532	0.8612
			H_2O	0.93038	0.519
		Trt D 1 vs Trt D 1	CS 2 vs CS 200	0.92467	0.7971
			CS 2 vs Cu+Man	1.0235	0.4087
			CS 200 vs Cu+Man	1.1969	0.0847
		Trt D 7 vs Trt D 7	CS 2 vs CS 200	1.276	0.0277
			CS 2 vs Cu+Man	1.1962	0.0279
			CS 200 vs Cu+Man	1.3997	0.0269
	2	D 1 vs H_2O D 1	CS 2	0.96014	0.6559

			CS 200	1.2531	0.0276
			Cu+Man	1.0876	0.2295
			RS	1.2929	0.0866
		D 7 vs H_2O D 7	CS 2	0.96085	0.6928
			CS 200	1.1149	0.1136
			Cu+Man	1.0977	0.0601
			RS	0.94253	0.6867
		D 1 vs No H_2O D 0	CS 2	1.0647	0.2297
			CS 200	1.5431	0.0304
			Cu+Man	1.073	0.196
			RS	1.4486	0.0297
			H_2O	1.1186	0.0845
		D 7 vs No H_2O D 0	CS 2	1.1776	0.0285
			CS 200	1.2826	0.0309
			Cu+Man	1.1732	0.0257
			RS	1.1912	0.0908
			H_2O	1.0729	0.1361
		D 1 vs D 7	CS 2	1.0389	0.2839
			CS 200	1.0228	0.4278
			Cu+Man	1.0594	0.2245
			RS	1.1224	0.1708
			H_2O	0.97739	0.5672
		Trt D 1 vs Trt D 1	CS 2 vs CS 200	1.3197	0.0303
			CS 2 vs Cu+Man	1.107	0.1762
			CS 200 vs Cu+Man	1.0832	0.2278
		Trt D 7 vs Trt D 7	CS 2 vs CS 200	1.2024	0.0301
			CS 2 vs Cu+Man	1.191	0.0303
			CS 200 vs Cu+Man	1.2543	0.0289
Gower excluding joint absences	1	D 1 vs H_2O D 1	CS 2	0.95947	0.712
			CS 200	1.1276	0.1451
			Cu+Man	0.92609	0.7375
			RS	1.1192	0.117
		D 7 vs H_2O D 7	CS 2	0.96996	0.6062

		CS 200	1.3164	0.0275
		Cu+Man	1.2177	0.0303
		RS	1.0599	0.1735
	D 1 vs No H_2O D 0	CS 2	0.92045	0.8838
		CS 200	1.1402	0.0849
		Cu+Man	1.0567	0.2904
		RS	1.1741	0.0288
		H_2O	0.97316	0.5122
	D 7 vs No H_2O D 0	CS 2	1.0166	0.3709
		CS 200	1.3301	0.0318
		Cu+Man	1.1602	0.0557
		RS	1.0511	0.3156
		H_2O	1.0146	0.3743
	D 1 vs D 7	CS 2	0.91615	0.9488
		CS 200	1.0062	0.3729
		Cu+Man	1.0428	0.2858
		RS	0.94549	0.8317
		H_2O	0.95318	0.6044
	Trt D 1 vs Trt D 1	CS 2 vs CS 200	0.91	0.882
		CS 2 vs Cu+Man	1.0605	0.2832
		CS 200 vs Cu+Man	1.1954	0.0583
	Trt D 7 vs Trt D 7	CS 2 vs CS 200	1.2096	0.0292
		CS 2 vs Cu+Man	1.1586	0.0833
		CS 200 vs Cu+Man	1.3257	0.0298
2	D 1 vs H_2O D 1	CS 2	0.95887	0.6279
		CS 200	1.2762	0.0275
		Cu+Man	1.1272	0.2213
		RS	1.3344	0.0859
	D 7 vs H_2O D 7	CS 2	0.94913	0.7146
		CS 200	1.1432	0.1098
		Cu+Man	1.1009	0.086
		RS	0.91817	0.7972
	D 1 vs No H_2O D 0	CS 2	1.0703	0.2299

			CS 200	1.6	0.0263
			Cu+Man	1.1292	0.2283
			RS	1.5122	0.0278
			H_2O	1.1217	0.1731
		D 7 vs No H_2O D 0	CS 2	1.2013	0.0322
			CS 200	1.3168	0.0297
			Cu+Man	1.1843	0.0288
			RS	1.1781	0.1137
			H_2O	1.0828	0.1493
		D 1 vs D 7	CS 2	1.0451	0.2655
			CS 200	1.0184	0.4538
			Cu+Man	1.109	0.2048
			RS	1.1764	0.1443
			H_2O	0.97666	0.5435
		Trt D 1 vs Trt D 1	CS 2 vs CS 200	1.348	0.0281
			CS 2 vs Cu+Man	1.1568	0.198
			CS 200 vs Cu+Man	1.0698	0.2864
		Trt D 7 vs Trt D 7	CS 2 vs CS 200	1.2383	0.0317
			CS 2 vs Cu+Man	1.212	0.0337
			CS 200 vs Cu+Man	1.3047	0.027
ModGower	1	D 1 vs H_2O D 1	CS 2	0.96402	0.8041
			CS 200	1.1586	0.1165
			Cu+Man	1.1353	0.1122
			RS	0.91711	0.8312
		D 7 vs H_2O D 7	CS 2	0.98925	0.491
			CS 200	1.3508	0.0309
			Cu+Man	1.2616	0.0523
			RS	1.0545	0.2812
		D 1 vs No H_2O D 0	CS 2	0.89527	0.9132
			CS 200	1.1357	0.0856
			Cu+Man	1.102	0.1666
			RS	1.2072	0.0287
			H_2O	0.99279	0.5114

	D 7 vs No H_2O D 0	CS 2	1.0344	0.3459
		CS 200	1.339	0.0305
		Cu+Man	1.1775	0.0549
		RS	1.0539	0.2861
		H_2O	1.0421	0.301
	D 1 vs D 7	CS 2	0.88275	0.9433
		CS 200	0.9726	0.5734
		Cu+Man	1.0174	0.4566
		RS	0.93035	0.8345
		H_2O	0.96988	0.5747
	Trt D 1 vs Trt D 1	CS 2 vs CS 200	0.85903	0.9116
		CS 2 vs Cu+Man	1.0792	0.1072
		CS 200 vs Cu+Man	1.1874	0.0525
	Trt D 7 vs Trt D 7	CS 2 vs CS 200	1.2106	0.0538
		CS 2 vs Cu+Man	1.1546	0.0861
		CS 200 vs Cu+Man	1.3188	0.0277
2	D 1 vs H_2O D 1	CS 2	0.98763	0.5783
		CS 200	1.1846	0.0299
		Cu+Man	1.074	0.1982
		RS	1.2689	0.0262
	D 7 vs H_2O D 7	CS 2	0.94	0.7644
		CS 200	1.109	0.1405
		Cu+Man	1.0472	0.2006
		RS	0.92093	0.8591
	D 1 vs No H_2O D 0	CS 2	1.0554	0.2224
		CS 200	1.4259	0.0315
		Cu+Man	1.0462	0.2187
		RS	1.414	0.0267
		H_2O	1.0888	0.1132
	D 7 vs No H_2O D 0	CS 2	1.1689	0.0298
		CS 200	1.2447	0.0303
		Cu+Man	1.1291	0.0283
		RS	1.1437	0.0859

			H_2O	1.0667	0.1115
		D 1 vs D 7	CS 2	1.0451	0.2284
			CS 200	1.0068	0.4539
			Cu+Man	1.0568	0.1938
			RS	1.1702	0.0873
			H_2O	0.96742	0.5987
		Trt D 1 vs Trt D 1	CS 2 vs CS 200	1.2534	0.029
			CS 2 vs Cu+Man	1.0805	0.1703
			CS 200 vs Cu+Man	1.0448	0.2259
		Trt D 7 vs Trt D 7	CS 2 vs CS 200	1.1899	0.0282
			CS 2 vs Cu+Man	1.1525	0.0295
			CS 200 vs Cu+Man	1.2522	0.0275
Jaccard	1	D 1 vs H_2O D 1	CS 2	2.318	0.3727
			CS 200	1.898	0.1984
			Cu+Man	1.6039	0.3659
			RS	0.57745	0.6239
		D 7 vs H_2O D 7	CS 2	0.048381	0.6877
			CS 200	1.2061	0.4606
			Cu+Man	1.0142	0.5403
			RS	Negative	
		D 1 vs No H_2O D 0	CS 2	19.485	0.0866
			CS 200	2.711	0.0566
			Cu+Man	2.1057	0.3697
			RS	3.7664	0.1155
			H_2O	2.6045	0.2805
		D 7 vs No H_2O D 0	CS 2	0.38049	0.6324
			CS 200	1.7024	0.3426
			Cu+Man	3.1924	0.2853
			RS	Negative	
			H_2O	2.0166	0.3145
		D 1 vs D 7	CS 2	1.3197	0.35
			CS 200	1.9996	0.0606
			Cu+Man	1.2087	0.5165

		RS	0.83117	0.6045
		H_2O	1.2085	0.5375
	Trt D 1 vs Trt D 1	CS 2 vs CS 200	4.1857	0.1743
		CS 2 vs Cu+Man	2.1641	0.2563
		CS 200 vs Cu+Man	1.0302	0.3164
	Trt D 7 vs Trt D 7	CS 2 vs CS 200	1.0372	0.4202
		CS 2 vs Cu+Man	1.2811	0.2812
		CS 200 vs Cu+Man	1.4134	0.366
2	D 1 vs H_2O D 1	CS 2	0.99427	0.515
		CS 200	1.1205	0.0626
		Cu+Man	1.0568	0.2275
		RS	1.2379	0.0325
	D 7 vs H_2O D 7	CS 2	0.93698	0.7745
		CS 200	1.0799	0.1108
		Cu+Man	1.011	0.3662
		RS	0.88445	0.9167
	D 1 vs No H_2O D 0	CS 2	1.0351	0.2873
		CS 200	1.3378	0.029
		Cu+Man	1.0358	0.2825
		RS	1.3699	0.0304
		H_2O	1.0659	0.2008
	D 7 vs No H_2O D 0	CS 2	1.1533	0.0273
		CS 200	1.1922	0.0309
		Cu+Man	1.0722	0.0867
		RS	1.091	0.1447
		H_2O	1.0638	0.1127
	D 1 vs D 7	CS 2	1.0518	0.1972
		CS 200	0.99616	0.5178
		Cu+Man	1.041	0.2932
		RS	1.1922	0.0571
		H_2O	0.96214	0.7425
	Trt D 1 vs Trt D 1	CS 2 vs CS 200	1.1916	0.0296
		CS 2 vs Cu+Man	1.0678	0.2087

			CS 200 vs Cu+Man	1.002	0.426
		Trt D 7 vs Trt D 7	CS 2 vs CS 200	1.1649	0.0275
			CS 2 vs Cu+Man	1.1157	0.0557
			CS 200 vs Cu+Man	1.2267	0.0265
Sorensen	1	D 1 vs H_2O D 1	CS 2	0.9423	0.6268
			CS 200	1.141	0.1421
			Cu+Man	0.89456	0.6842
			RS	1.1182	0.2013
		D 7 vs H_2O D 7	CS 2	0.95468	0.5764
			CS 200	1.4337	0.0305
			Cu+Man	1.2599	0.0272
			RS	1.0776	0.1715
		D 1 vs No H_2O D 0	CS 2	0.89976	0.7728
			CS 200	1.1548	0.1415
			Cu+Man	1.044	0.3175
			RS	1.1801	0.0853
			H_2O	0.94847	0.4553
		D 7 vs No H_2O D 0	CS 2	0.99897	0.3794
			CS 200	1.4498	0.0286
			Cu+Man	1.2156	0.0572
			RS	1.065	0.2863
			H_2O	0.98443	0.425
		D 1 vs D 7	CS 2	0.91914	0.7424
			CS 200	1.0554	0.3077
			Cu+Man	1.0713	0.2293
			RS	0.93462	0.9172
			H_2O	0.92948	0.5191
		Trt D 1 vs Trt D 1	CS 2 vs CS 200	0.89582	0.8617
			CS 2 vs Cu+Man	1.04	0.3451
			CS 200 vs Cu+Man	1.2393	0.0583
		Trt D 7 vs Trt D 7	CS 2 vs CS 200	1.2974	0.0274
			CS 2 vs Cu+Man	1.213	0.026

		CS 200 vs Cu+Man	1.45	0.03
2	D 1 vs H_2O D 1	CS 2	0.98989	0.5077
		CS 200	1.1515	0.0587
		Cu+Man	1.0808	0.2209
		RS	1.2958	0.0288
	D 7 vs H_2O D 7	CS 2	0.92556	0.7736
		CS 200	1.1008	0.1441
		Cu+Man	1.0176	0.337
		RS	0.86603	0.9177
	D 1 vs No H_2O D 0	CS 2	1.0413	0.3477
		CS 200	1.4189	0.0263
		Cu+Man	1.0882	0.2291
		RS	1.4672	0.0308
		H_2O	1.0815	0.2031
	D 7 vs No H_2O D 0	CS 2	1.1863	0.0259
		CS 200	1.2388	0.028
		Cu+Man	1.0908	0.0831
		RS	1.1139	0.1446
		H_2O	1.0768	0.1169
	D 1 vs D 7	CS 2	1.0598	0.219
		CS 200	0.99929	0.4855
		Cu+Man	1.0768	0.1776
		RS	1.2437	0.0552
		H_2O	0.95351	0.742
	Trt D 1 vs Trt D 1	CS 2 vs CS 200	1.2362	0.0281
		CS 2 vs Cu+Man	1.0967	0.2595
		CS 200 vs Cu+Man	0.97363	0.514
	Trt D 7 vs Trt D 7	CS 2 vs CS 200	1.2012	0.0299
		CS 2 vs Cu+Man	1.1402	0.0559
		CS 200 vs Cu+Man	1.28	0.0277

[a.] All data was transformed at the 4th square root unless otherwise noted.

[b.] Trial 1 and trial 2 were conducted April and December 2016, respectively.

[c.] Comparisons were abbreviated as treatment = Trt, Days post application = D

[d.] Treatments were abbreviated as core-shell copper nanomaterials = CS applied at two different concentrations 2 µg/ml = 2, and 200 µg/ml = 200, copper-mancozeb = Cu+Man, water control = H_2O, inoculated with *Ralstonia solanacearum* = RS

[e.] Each analysis consisted of 9999 permutations.

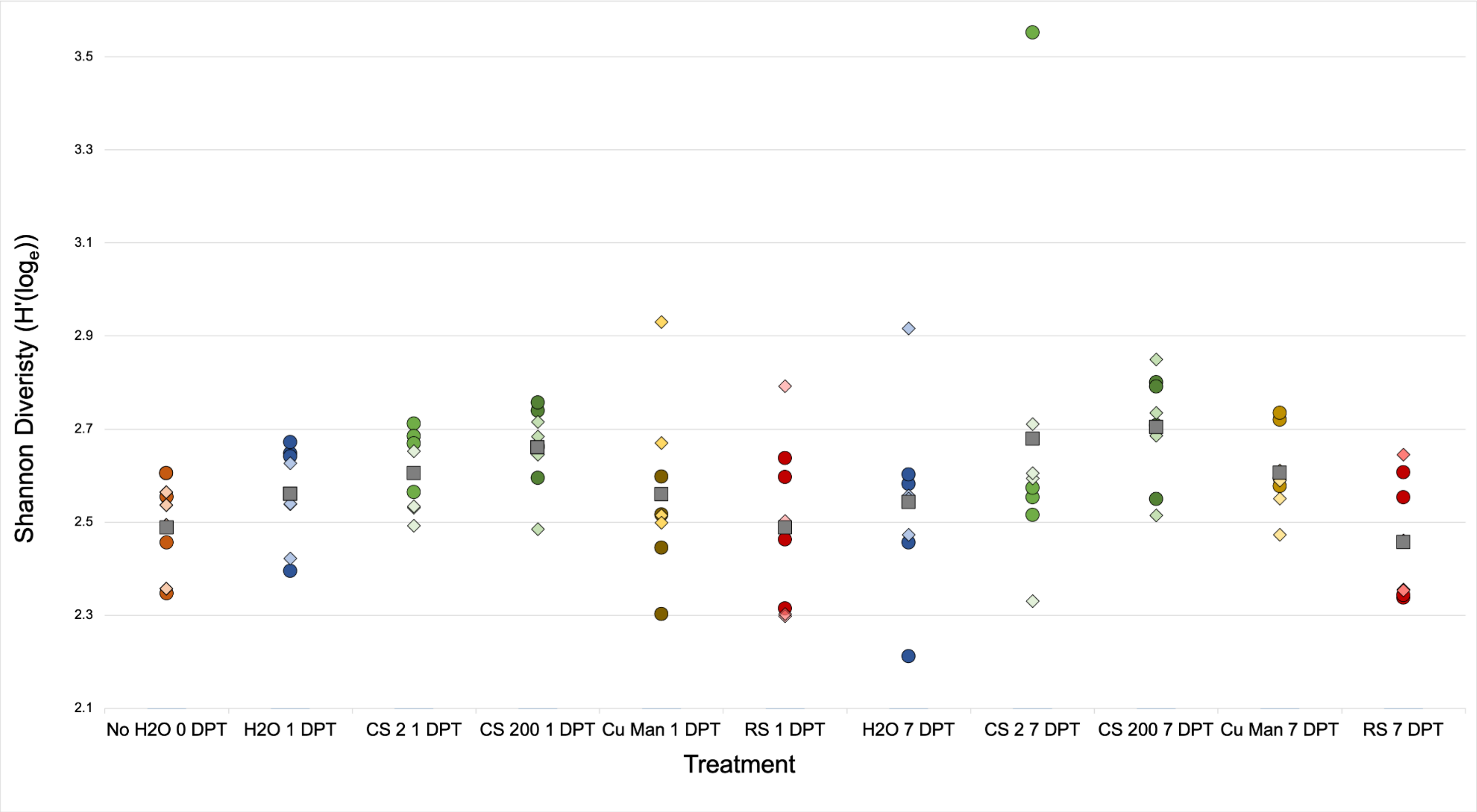

Fig. S1. The effect of metallic copper at 2 and 200 µg/ml from core-shell silica copper (CS 2 (the light green) and CS 200 (dark green), respectively, copper-mancozeb (Cu+Man (yellow)), *Ralstonia solanacearum* (RS (red)) at $5x10^8$ CFU/ml, and water (H_2O (blue)) compared to the nontreated control (No H_2O at 0 DPT (orange)) bacterial diversity in the soil microbiome in the first and second growth chamber experiments (Trial 1 (represented by the darker color and circles) and Trial 2 (represented in the lighter colors a[illegible] A [illegible]iamonds), respectively). Samples were collected at 1- and 7-days post treatment (DPT).

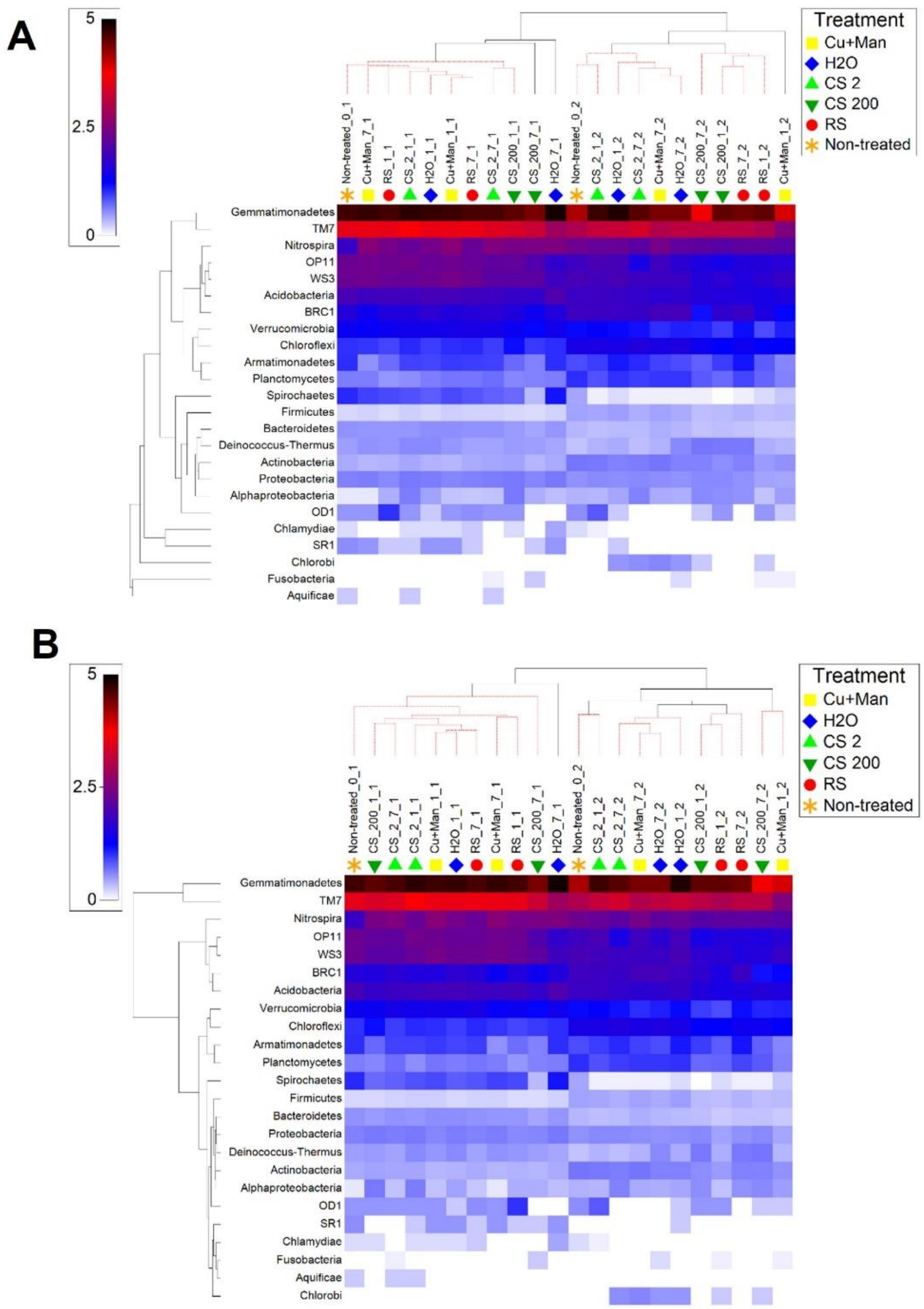

Fig. S2. Prevalence of bacterial phyla in soil, (non-treated, orange) and following treatment with metallic copper at 2 and 200 µg/ml from core-shell silica copper (CS 2 (the light green triangle) and CS 200 (dark green triangle), respectively, copper-mancozeb (Cu+Man (yellow)), *Ralstonia solanacearum* (RS (red)) at 5×10^8 CFU/ml, and water (H_2O (blue)). The sample labels first list the treatment, the day post treatment (0, 1, or 7) and then the trial (1 or 2, where 1 was conducted in the spring and 2 was conducted in the winter). The displayed samples are the average of four replications. The black lines in the samples' clade represent a statistical significant difference at $\alpha = 0.05$ as determined by SIMPROF. Clades were created by data transformed to the 4th root and analyzed by Bray-Curtis (**A**) or Euclidean (**B**).

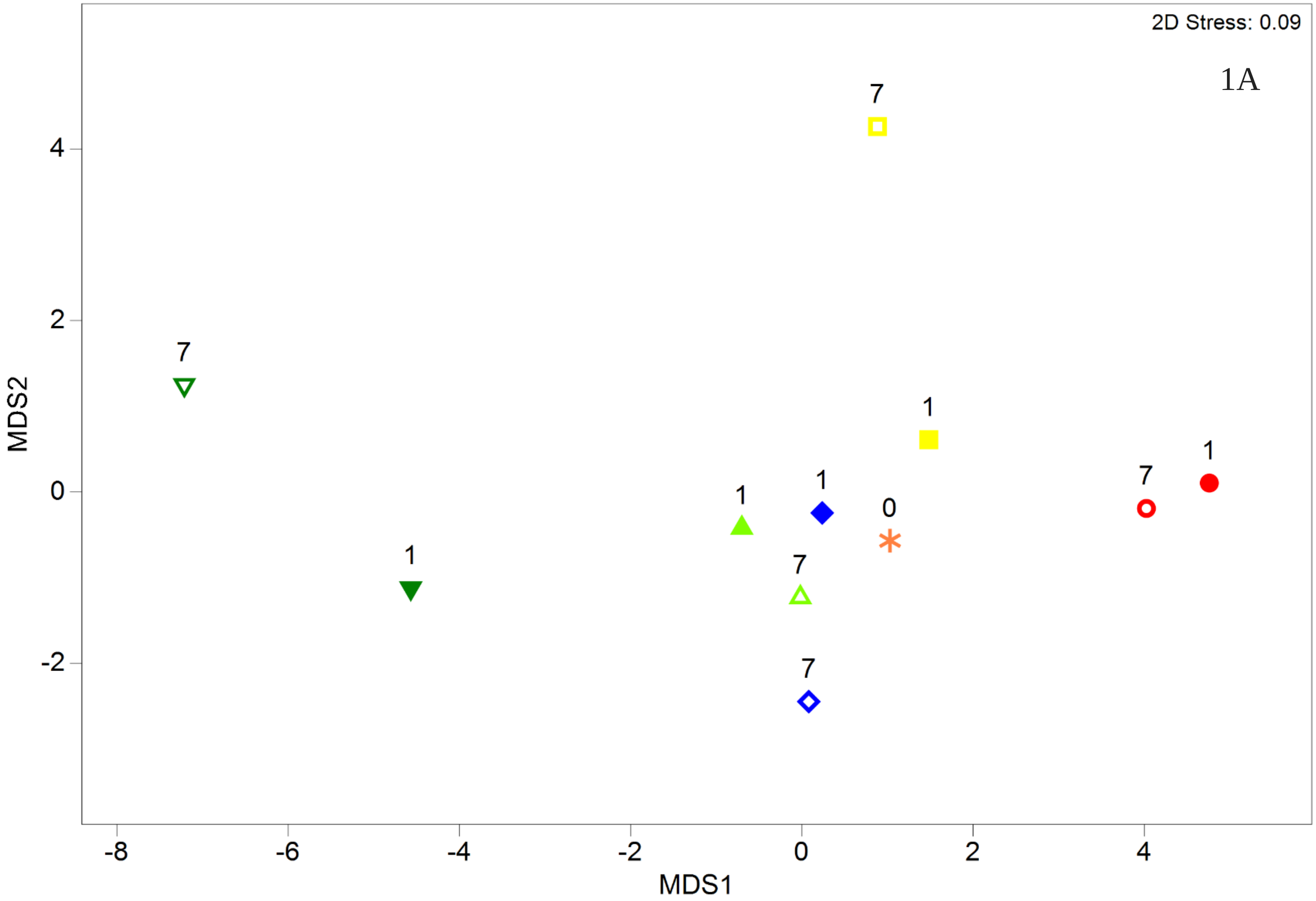
2D Stress: 0.09
1A
MDS2
MDS1
4
2
0
-2
-8
-6
-4
-2
0
2
4
7
7
1
1
1
7
1
1
0
7
7
7

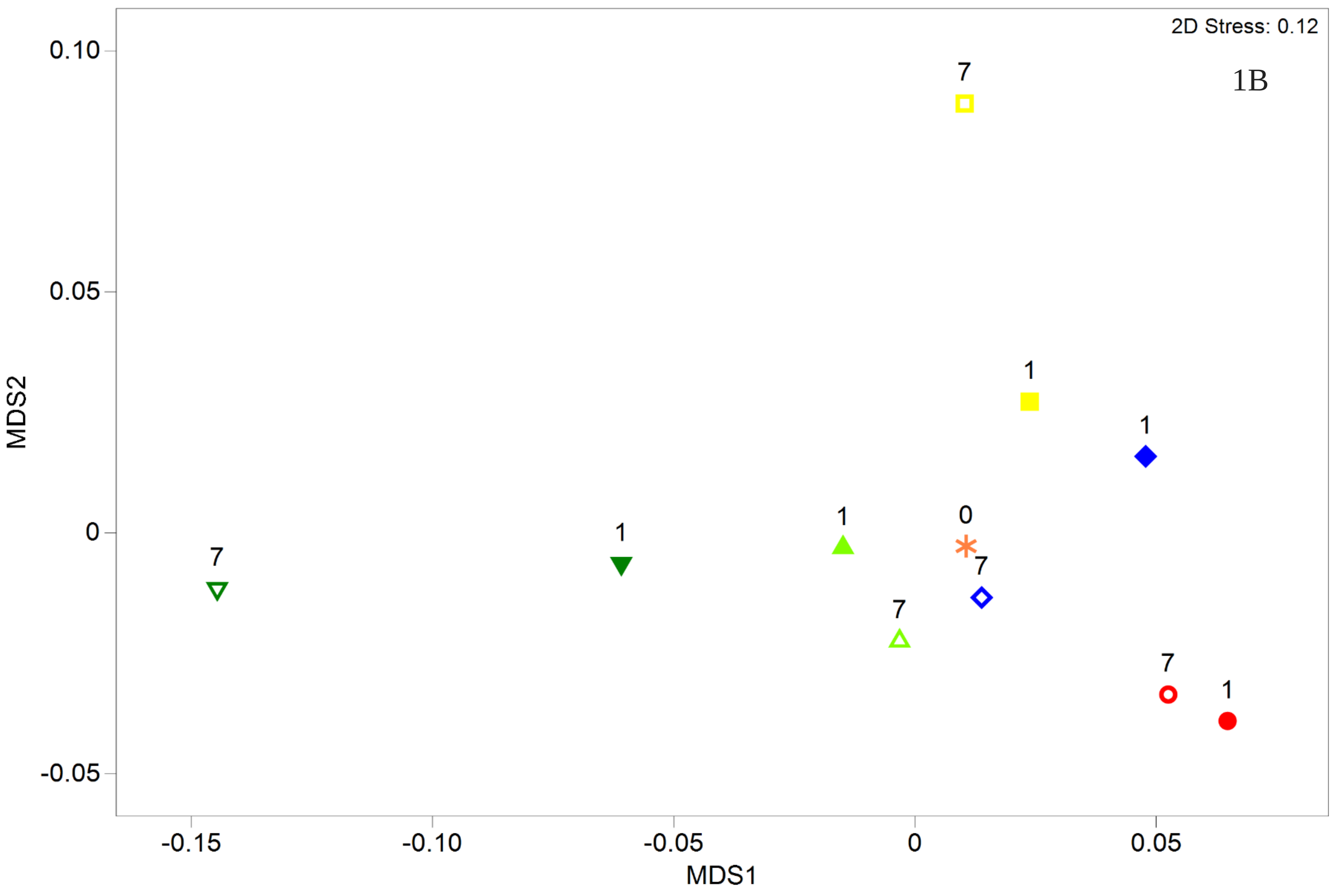
2D Stress: 0.12
1B
0.10
0.05
0
-0.05
MDS2
-0.15
-0.10
-0.05
0
0.05
MDS1
7
1
1
0
1
1
7
7
7
7
1

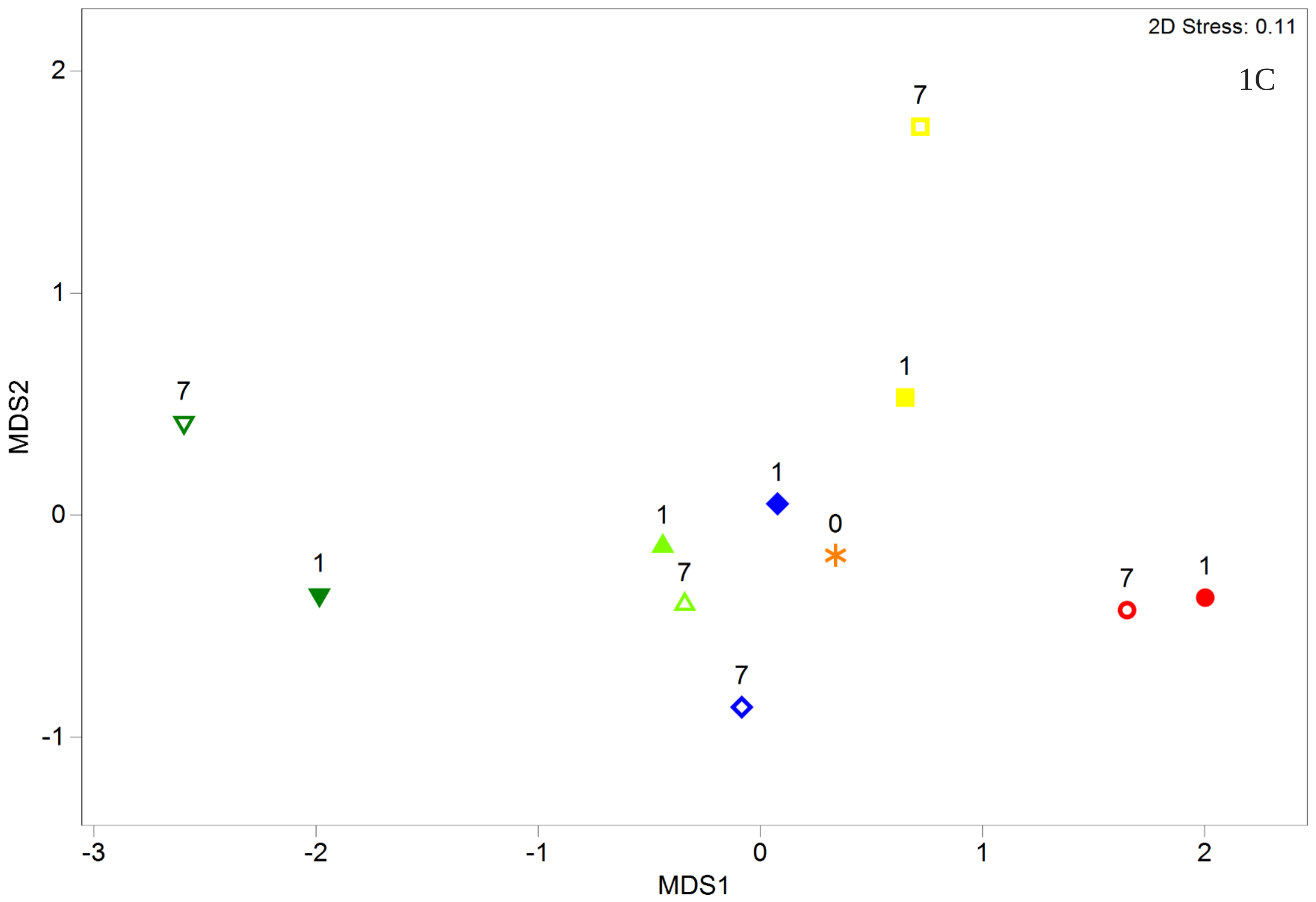

2D Stress: 0.11
1C
7
1
7
1
1
0
1
7
7
1
7
MDS2
MDS1
2
1
0
-1
-3
-2
-1
0
1
2

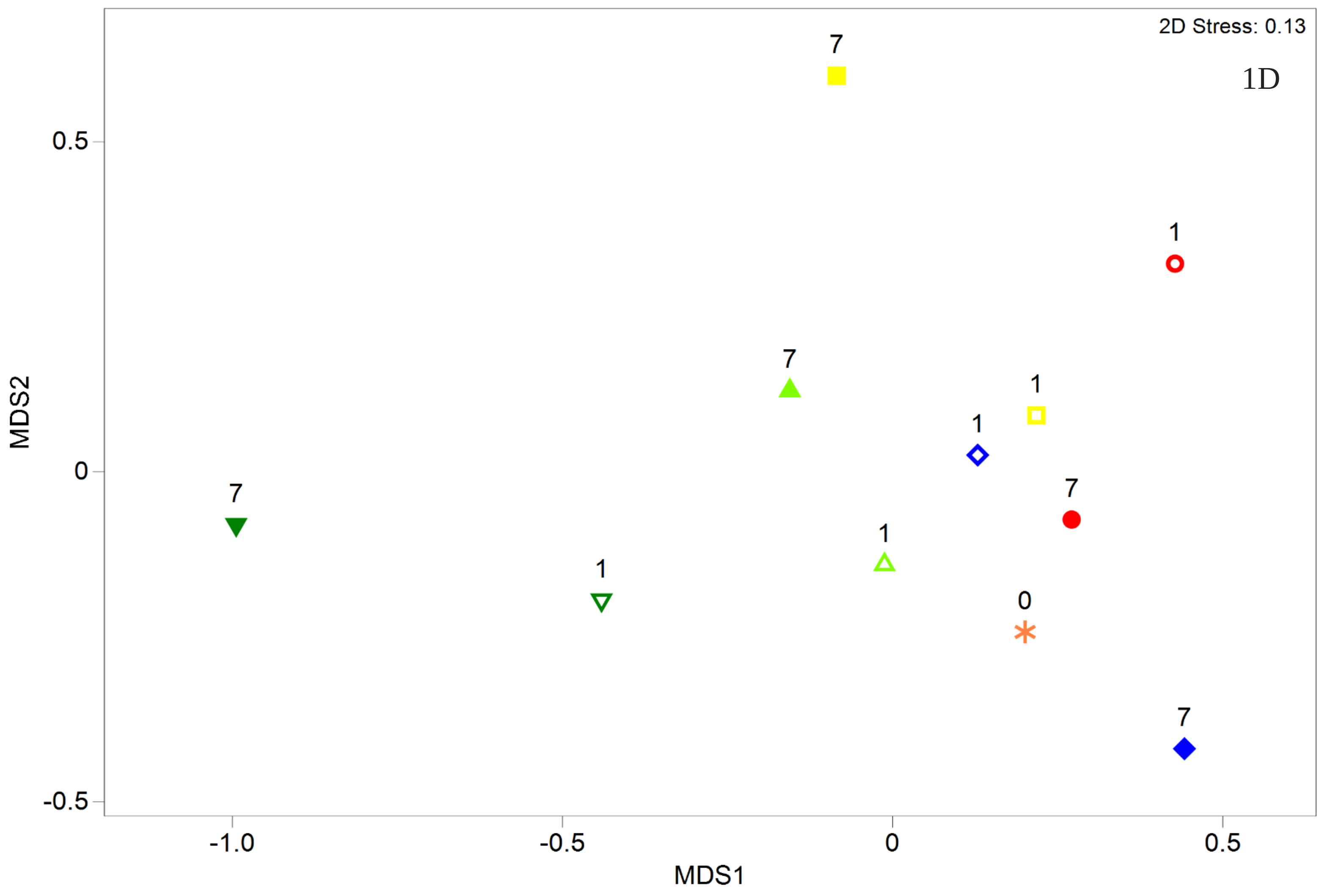
2D Stress: 0.13
1D
0.5
0
-0.5
-1.0
-0.5
0
0.5
MDS1
MDS2
7
7
7
7
7
7
1
1
1
1
1
1
0

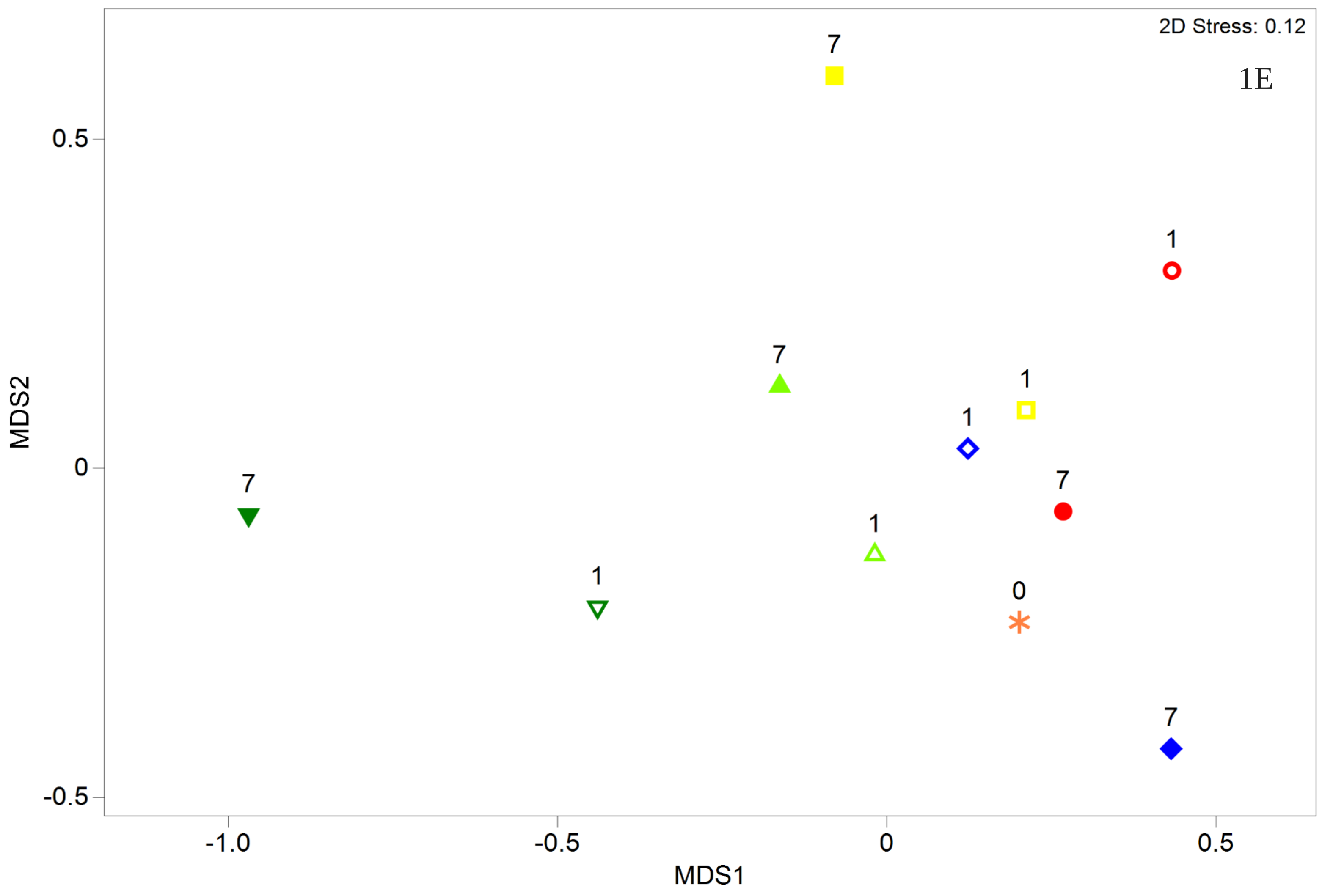

2D Stress: 0.12
1E
7
1
7
1
1
7
7
1
1
0
7
0.5
0
-0.5
MDS2
-1.0
-0.5
0
0.5
MDS1

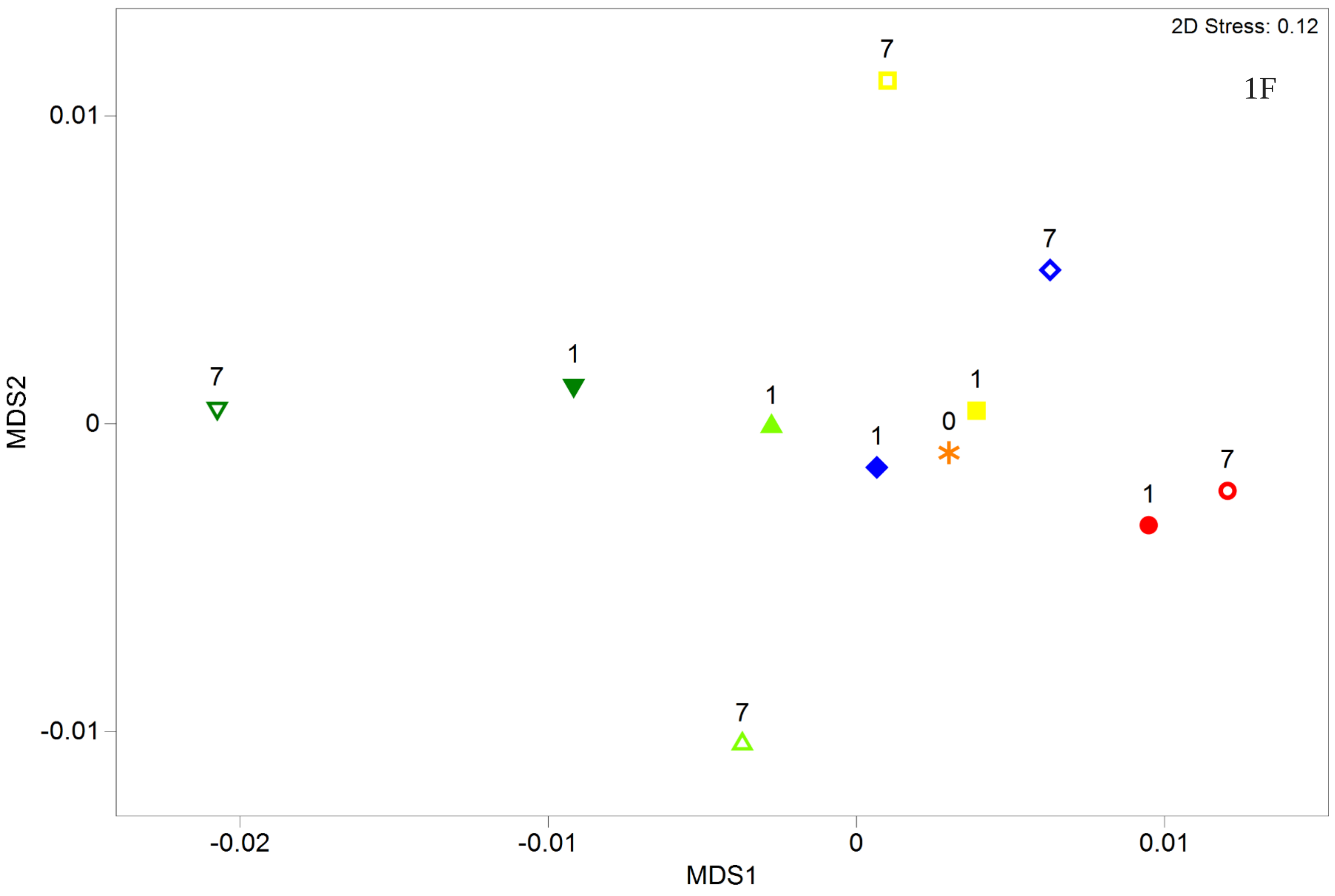
2D Stress: 0.12
1F
MDS2
MDS1
0.01
0
-0.01
-0.02
-0.01
0
0.01
7
7
1
1
1
1
0
7
1
7
7

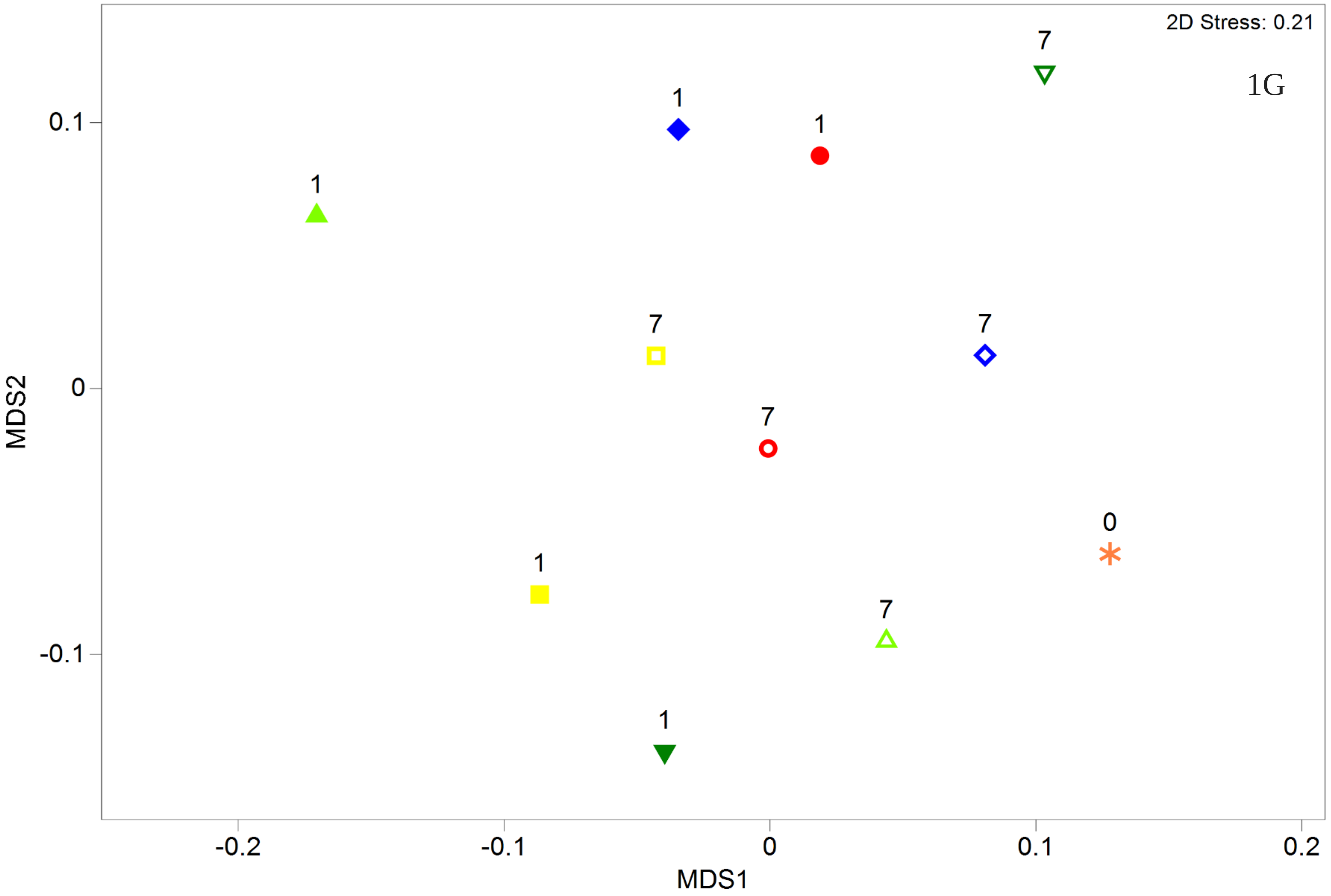
2D Stress: 0.21
1G
MDS1
MDS2
0.1
0
-0.1
-0.2
-0.1
0
0.1
0.2
1
1
1
1
1
7
7
7
7
7
0

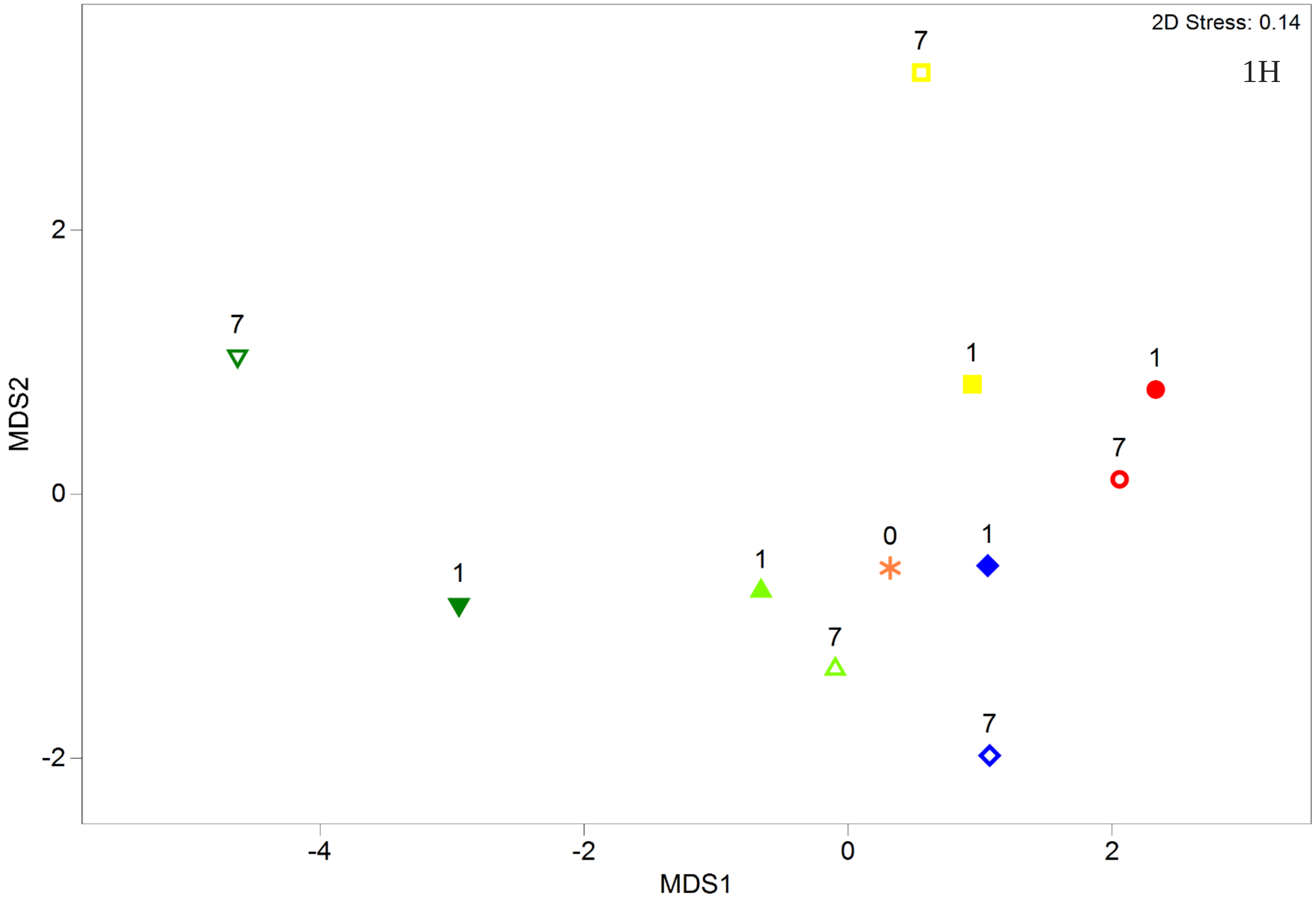

2D Stress: 0.14
1H
7
7
1
1
7
0
1
1
1
7
7
MDS2
MDS1
2
0
-2
-4
-2
0
2

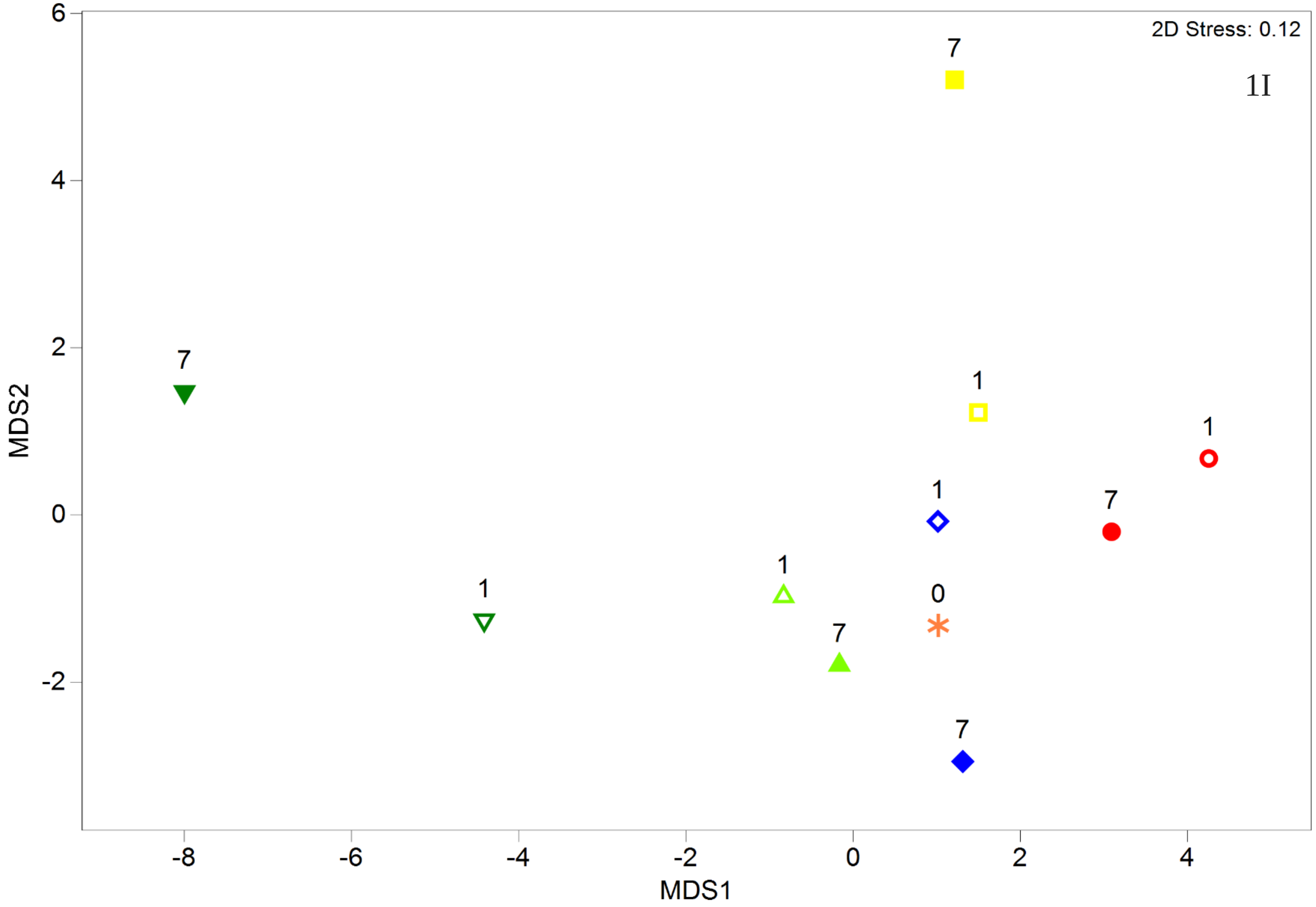
2D Stress: 0.12
1I
7
7
1
1
1
7
1
1
0
7
7
MDS2
6
4
2
0
-2
-8
-6
-4
-2
0
2
4
MDS1

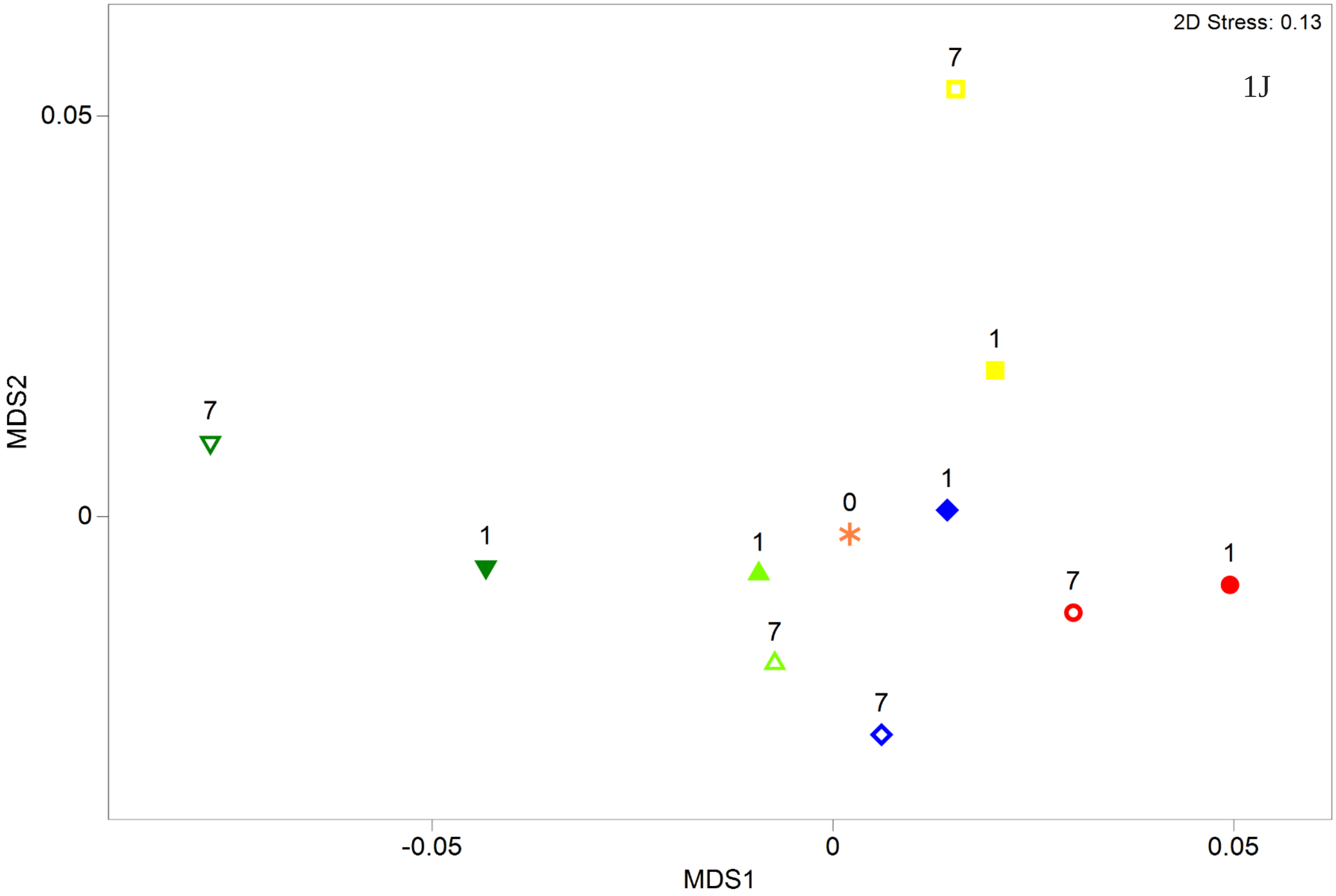

2D Stress: 0.13
1J
7
1
7
1
0
1
1
7
1
7
7
0.05
0
MDS2
-0.05
0
0.05
MDS1

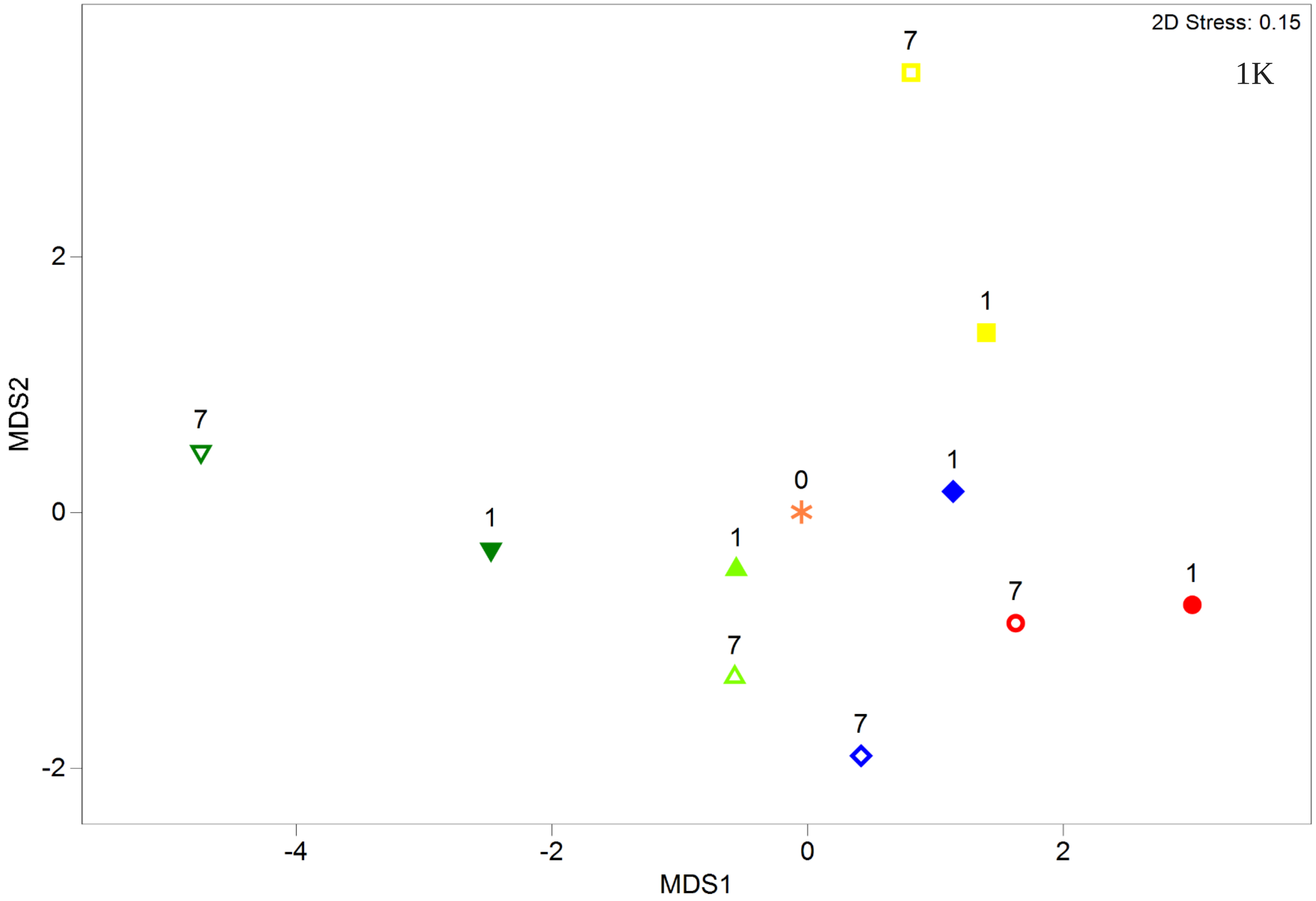
2D Stress: 0.15
1K
7
1
7
1
0
1
1
7
1
7
7
MDS2
2
0
-2
-4
-2
0
2
MDS1

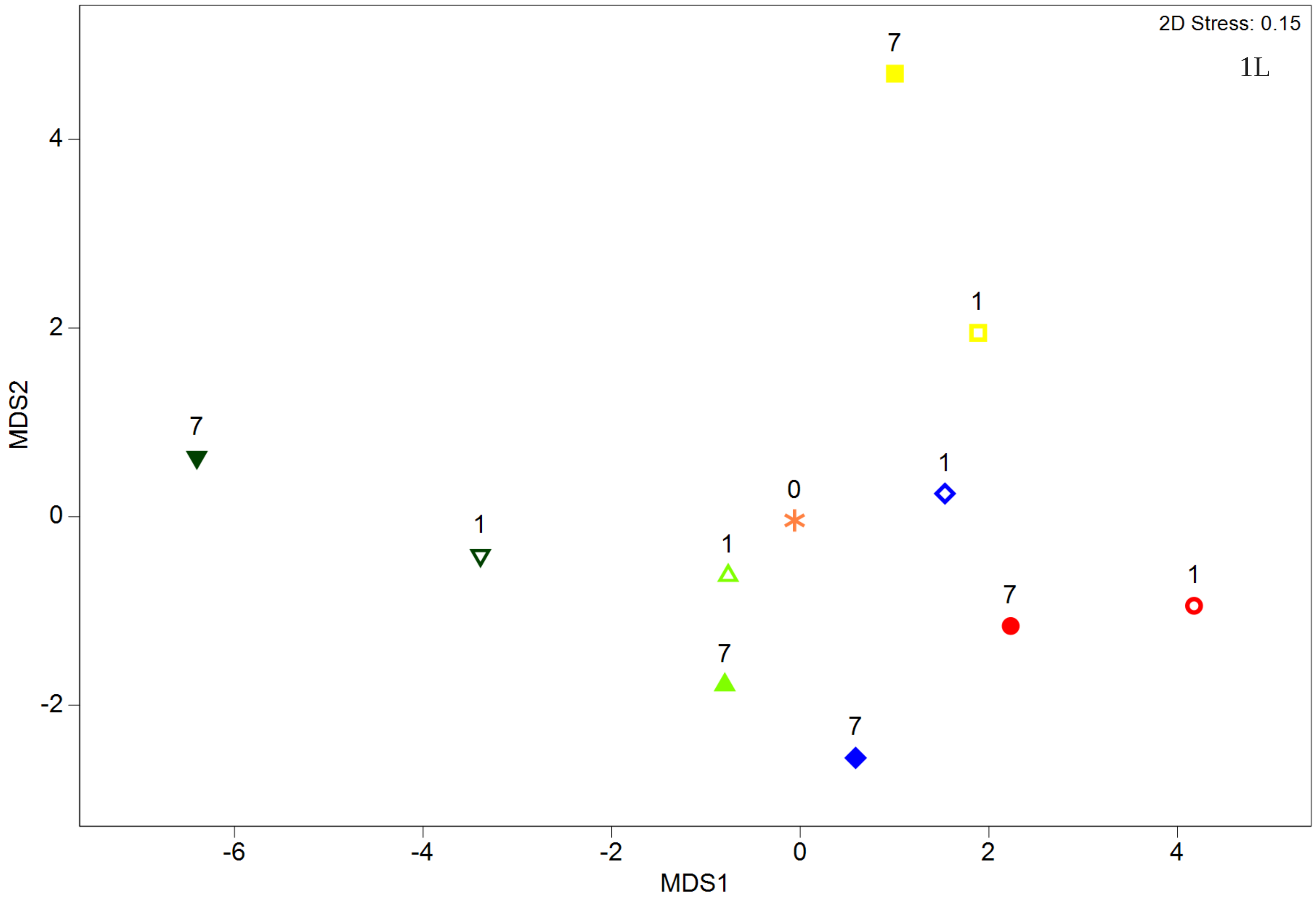

2D Stress: 0.15
1L
MDS2
MDS1
4
2
0
-2
-6
-4
-2
0
2
4
7
1
7
1
1
0
1
1
7
7
7

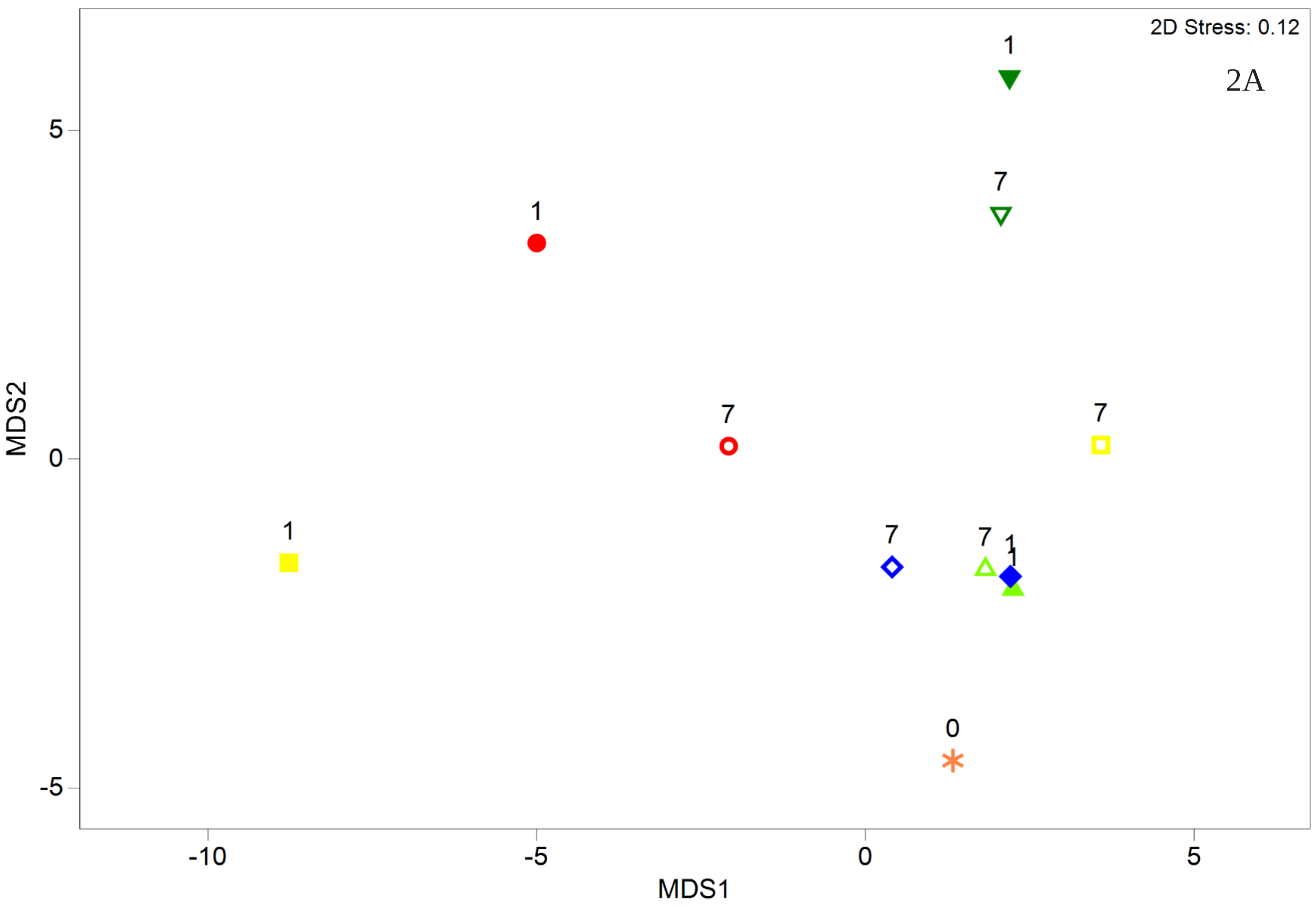

2D Stress: 0.12
2A
1
7
1
7
7
1
7
7
1
1
0
5
0
-5
-10
-5
0
5
MDS1
MDS2

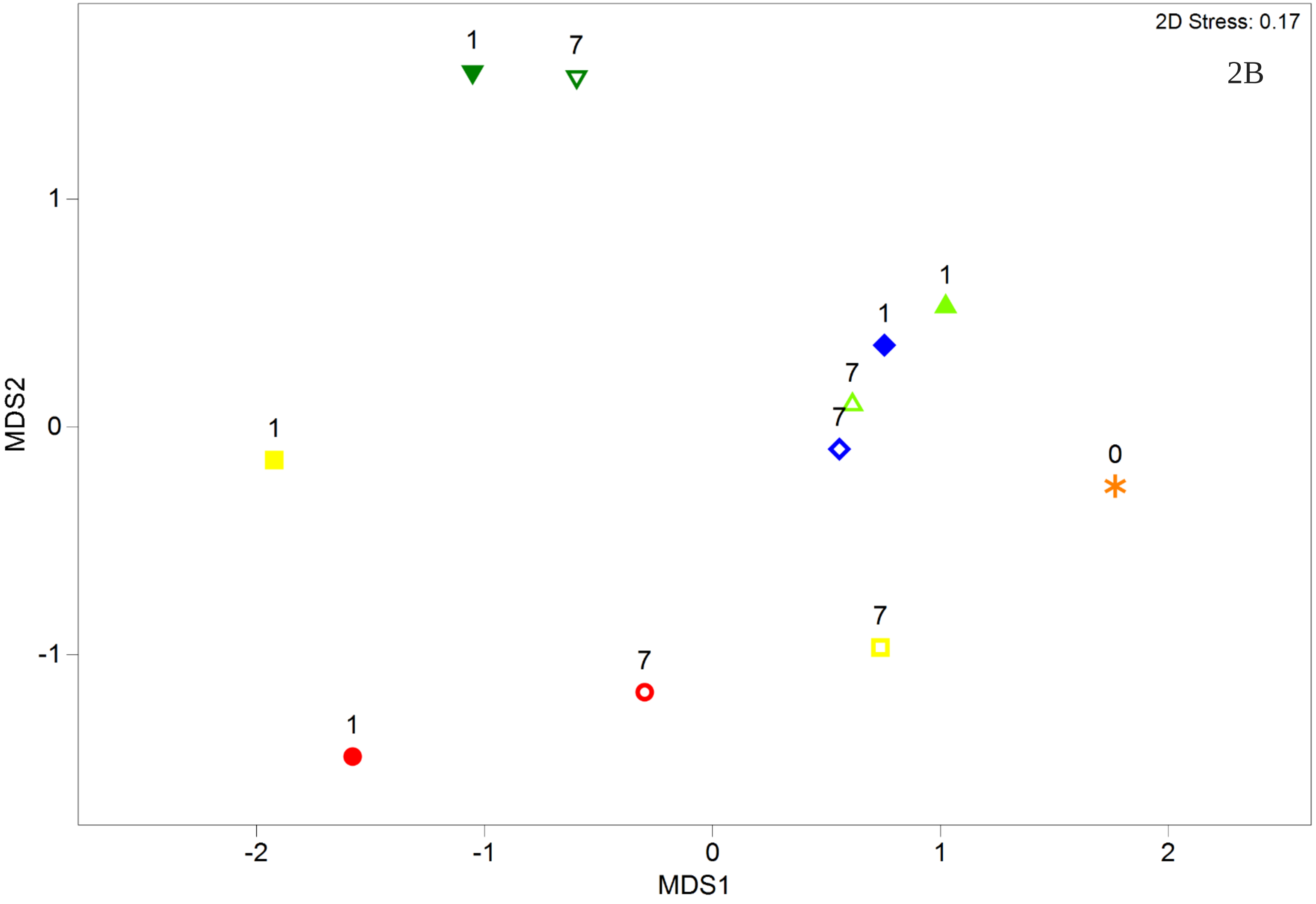

2D Stress: 0.17
2B
MDS2
MDS1
1
0
-1
-2
2
7

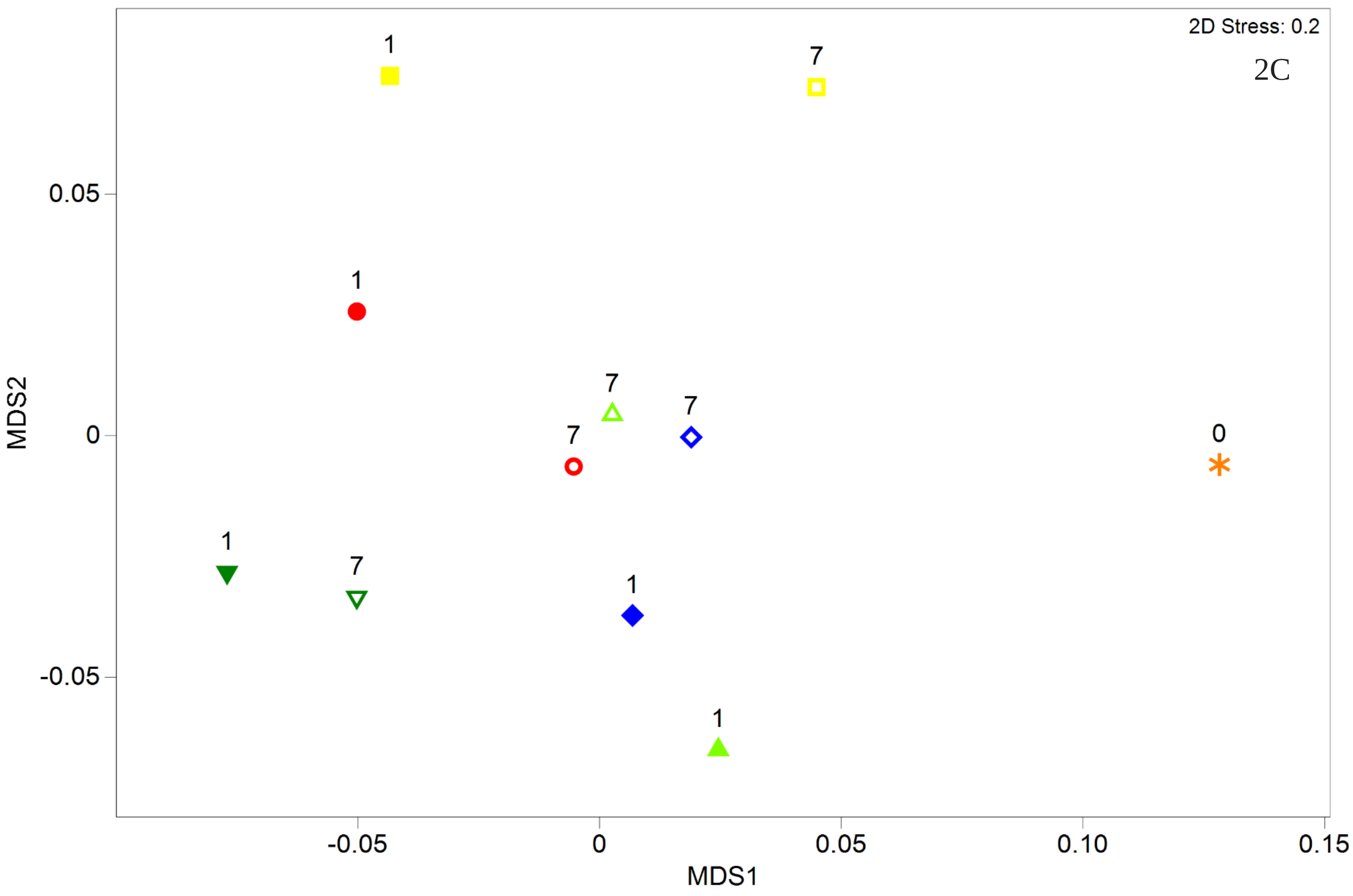

2D Stress: 0.2
2C
1
7
0.05
1
7
7
7
0
0
1
7
1
-0.05
1
-0.05
0
0.05
0.10
0.15
MDS1
MDS2

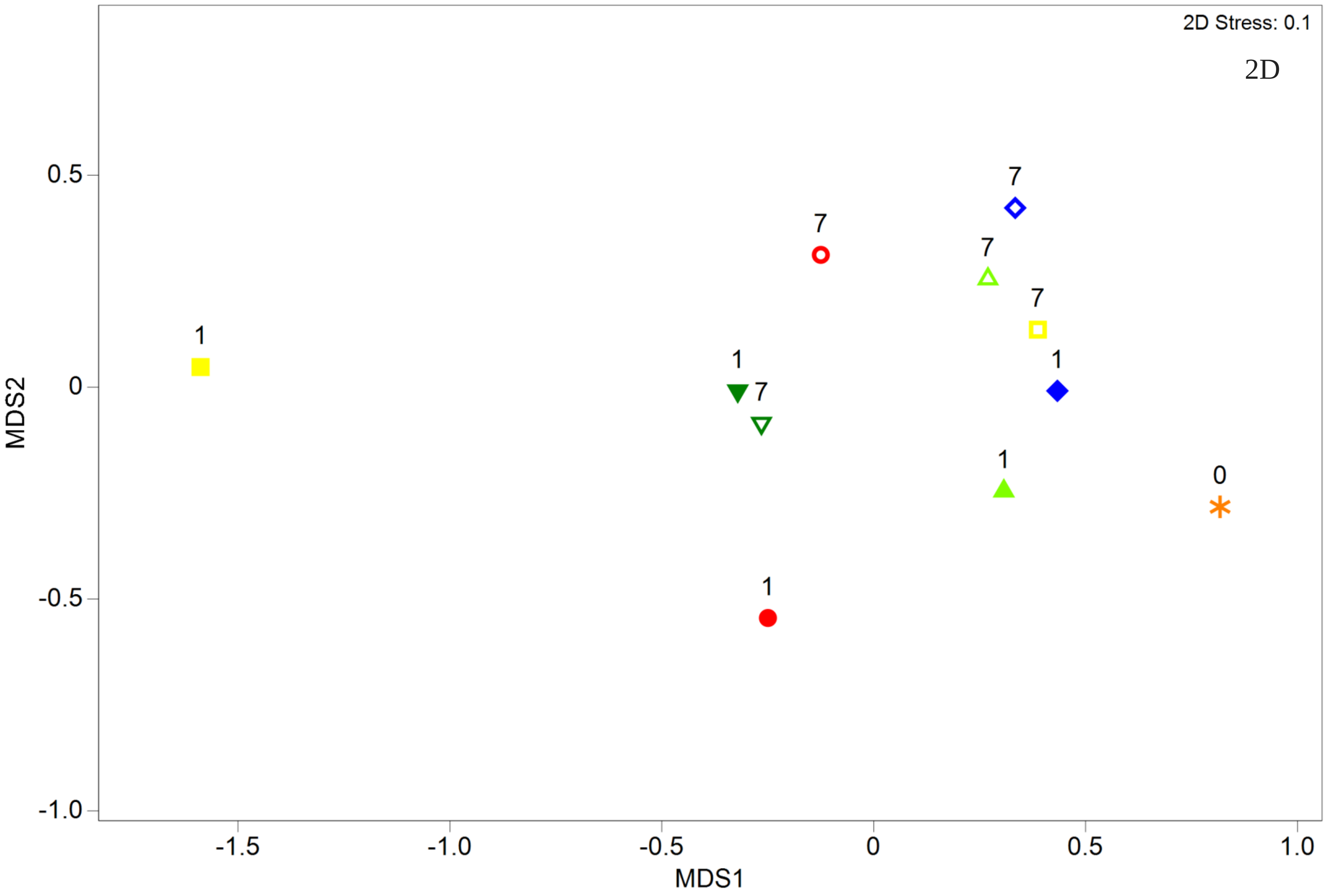
2D Stress: 0.1
2D
MDS2
MDS1
0.5
0
-0.5
-1.0
-1.5
-1.0
-0.5
0
0.5
1.0
7
7
7
7
1
1
7
1
1
0
1

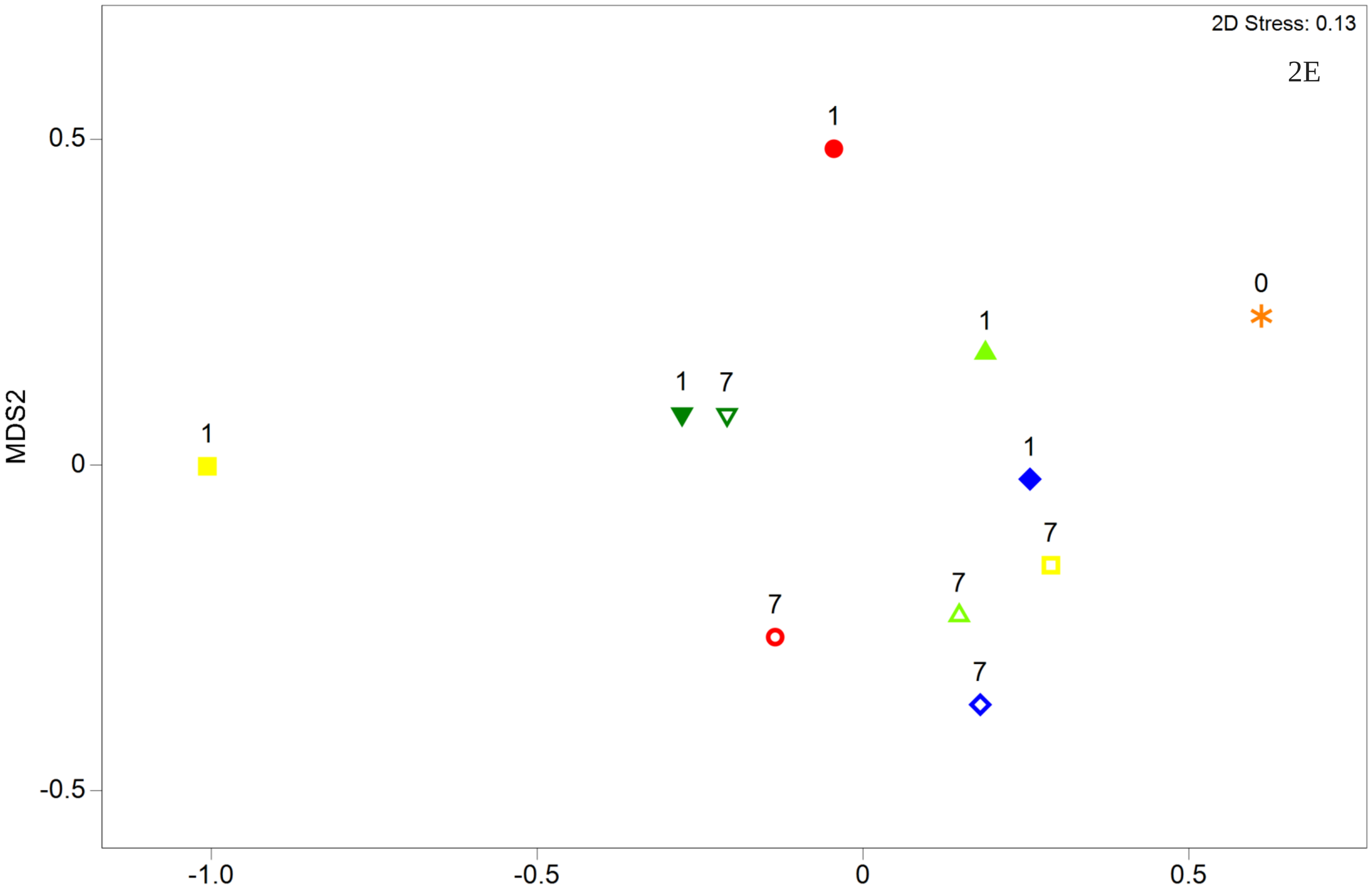
2D Stress: 0.13
2E
MDS2
0.5
0
-0.5
-1.0
-0.5
0
0.5
1
1
1
7
1
0
1
1
7
7
7
7
7

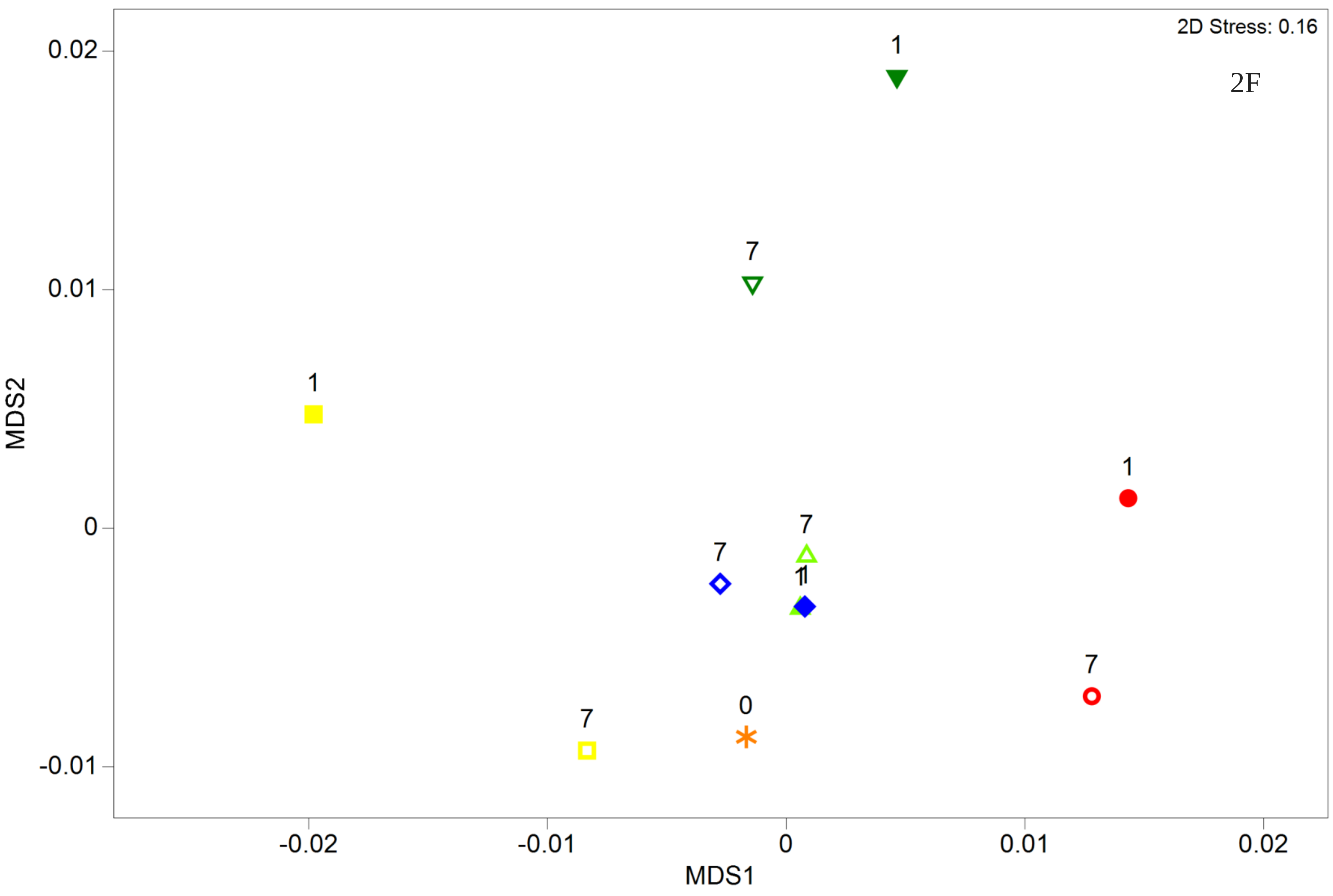
2D Stress: 0.16
2F
MDS2
MDS1
0.02
0.01
0
-0.01
-0.02
-0.01
0
0.01
0.02
1
7
1
1
7
7
1
1
7
7
0

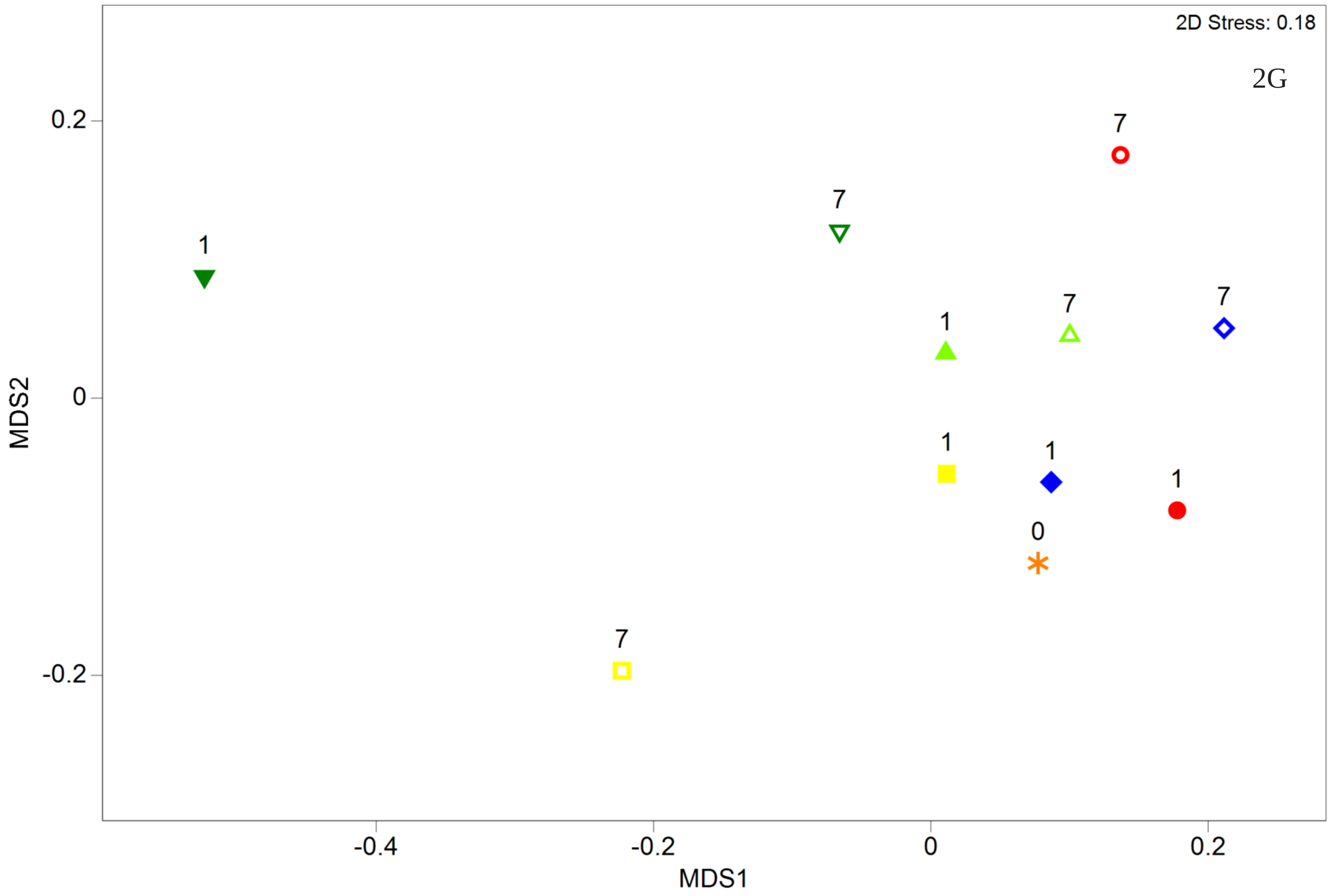

2D Stress: 0.18
2G
0.2
0
-0.2
MDS2
-0.4
-0.2
0
0.2
MDS1
1
7
7
1
7
7
1
1
1
1
0
7

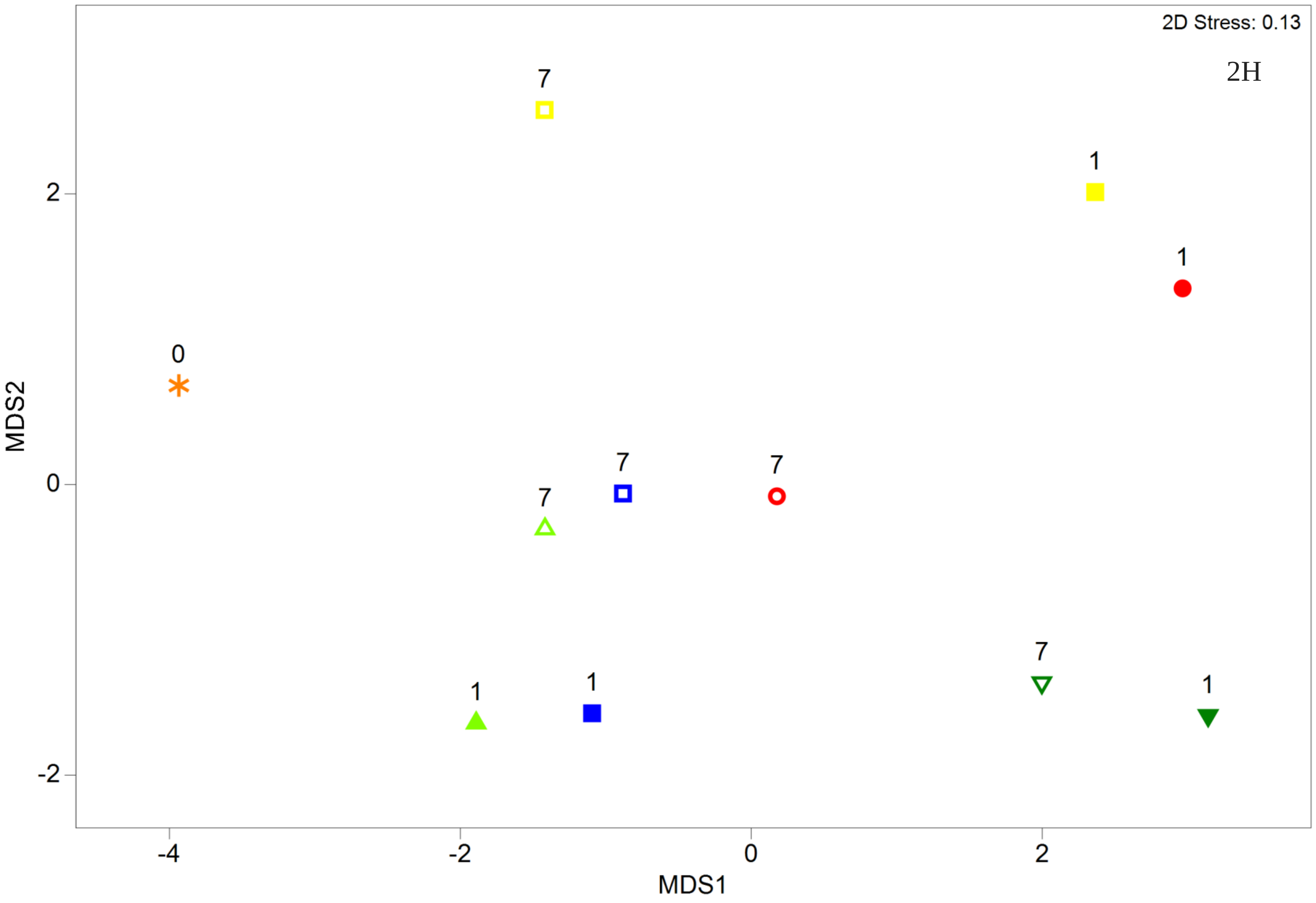
2D Stress: 0.13
2H
MDS2
MDS1
2
0
-2
-4
-2
0
2
7
1
1
0
7
7
7
7
1
1
1
1

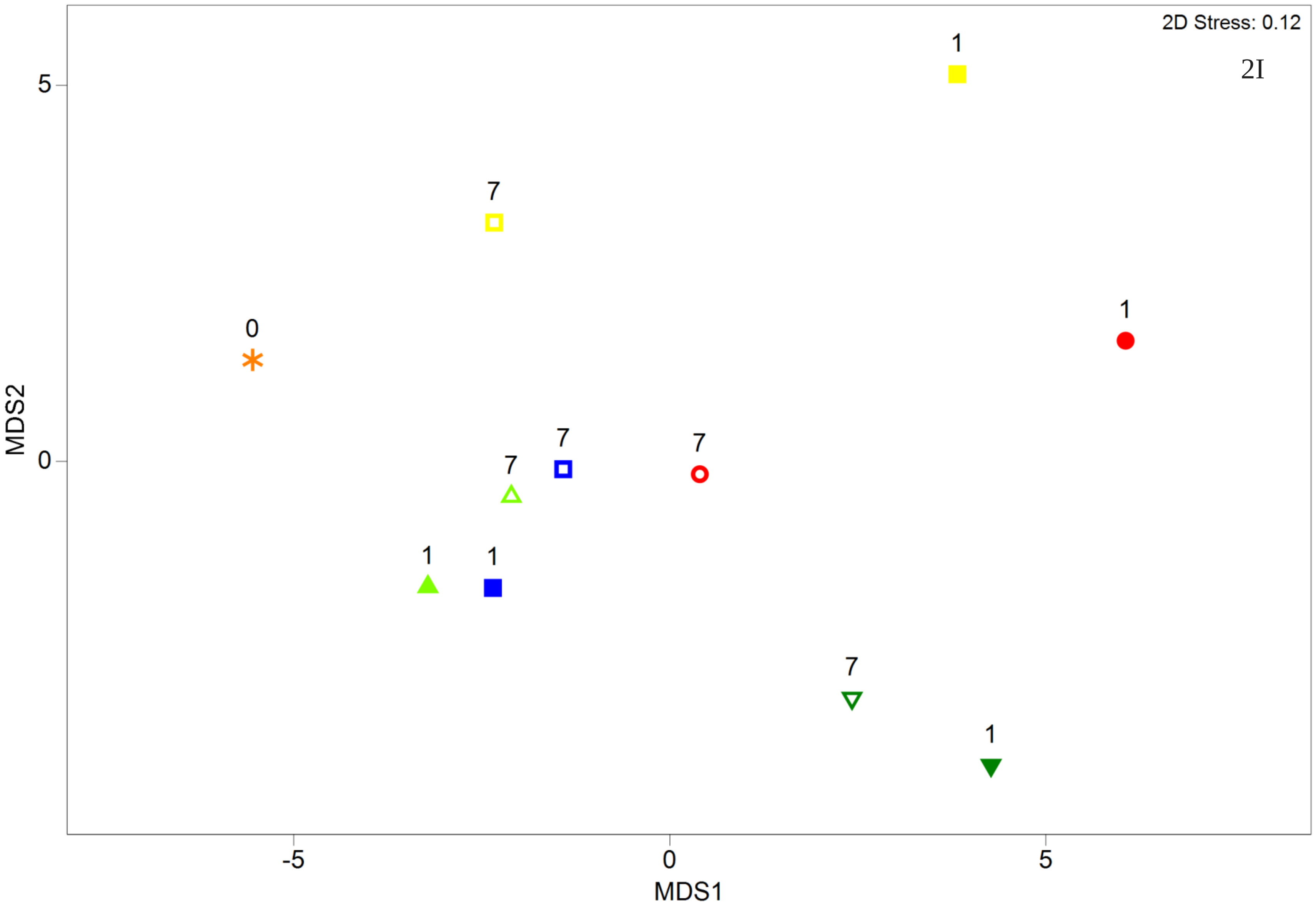
2D Stress: 0.12
2I
MDS2
MDS1
5
0
-5
0
5
1
7
0
1
7
7
7
1
1
7
1

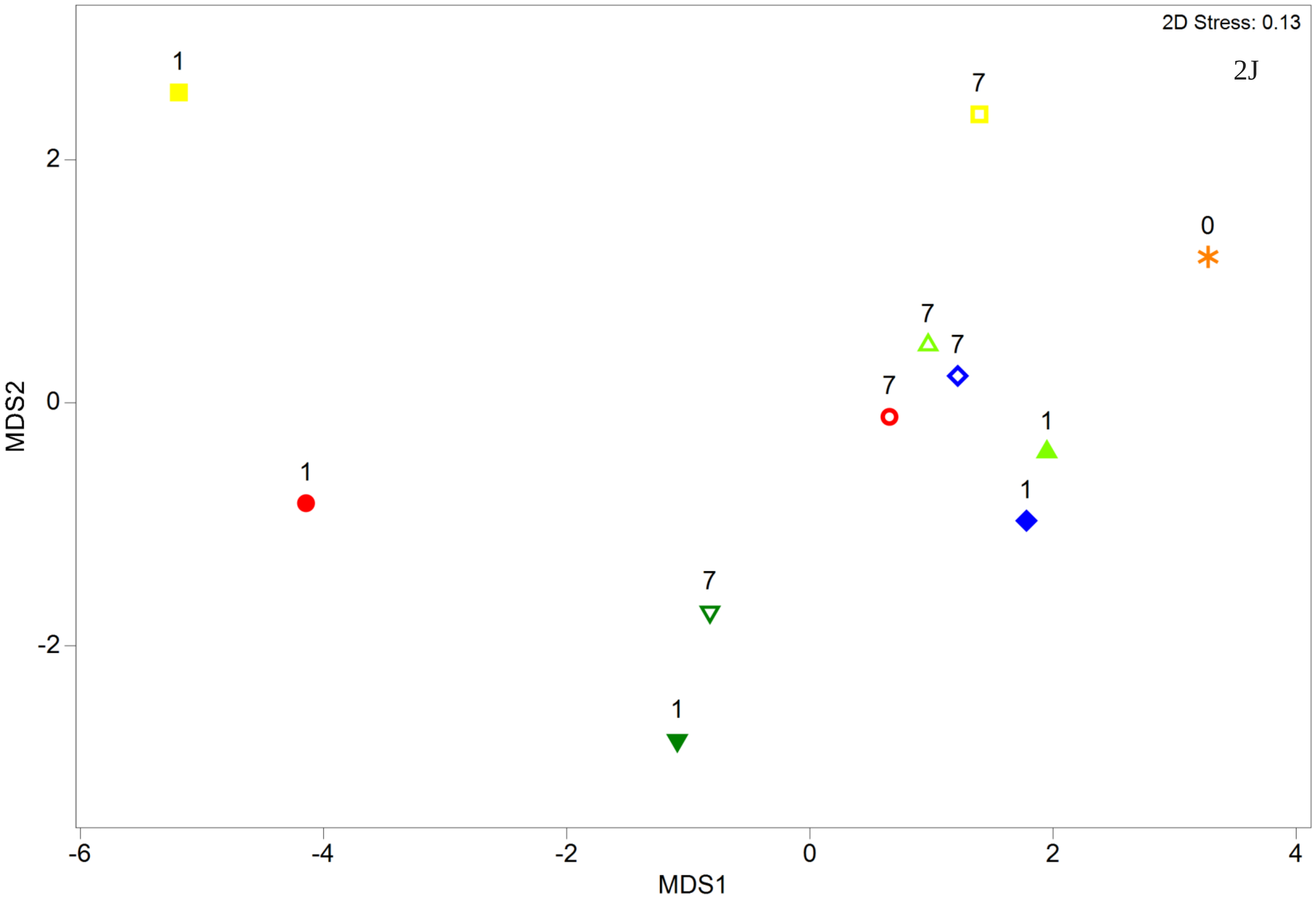
2D Stress: 0.13
2J
1
7
0
7
7
7
1
1
1
7
1
2
0
-2
-6
-4
-2
0
2
4
MDS1
MDS2

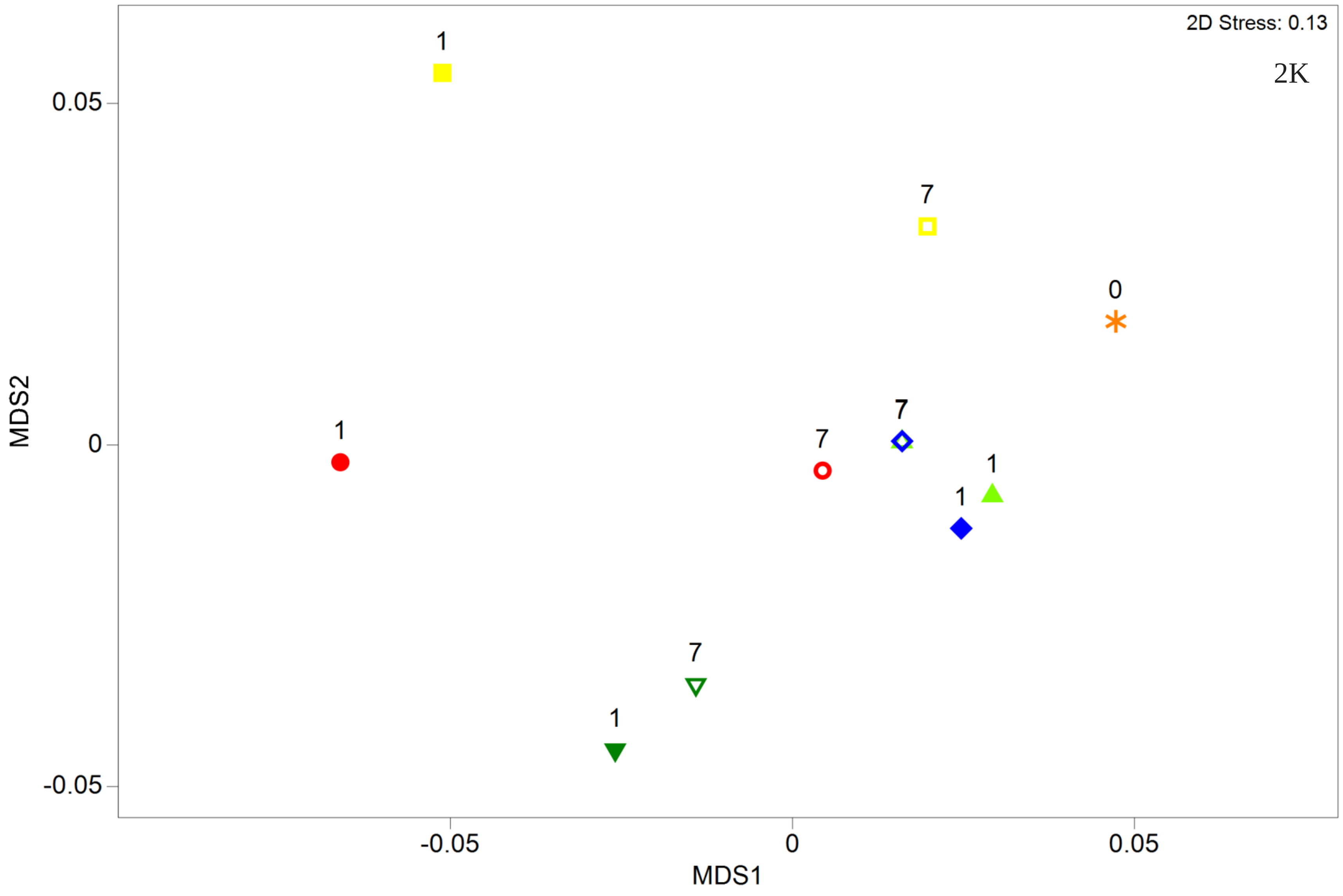

2D Stress: 0.13
2K
1
7
0
1
7
7
1
1
7
1
0.05
0
-0.05
-0.05
0
0.05
MDS1
MDS2

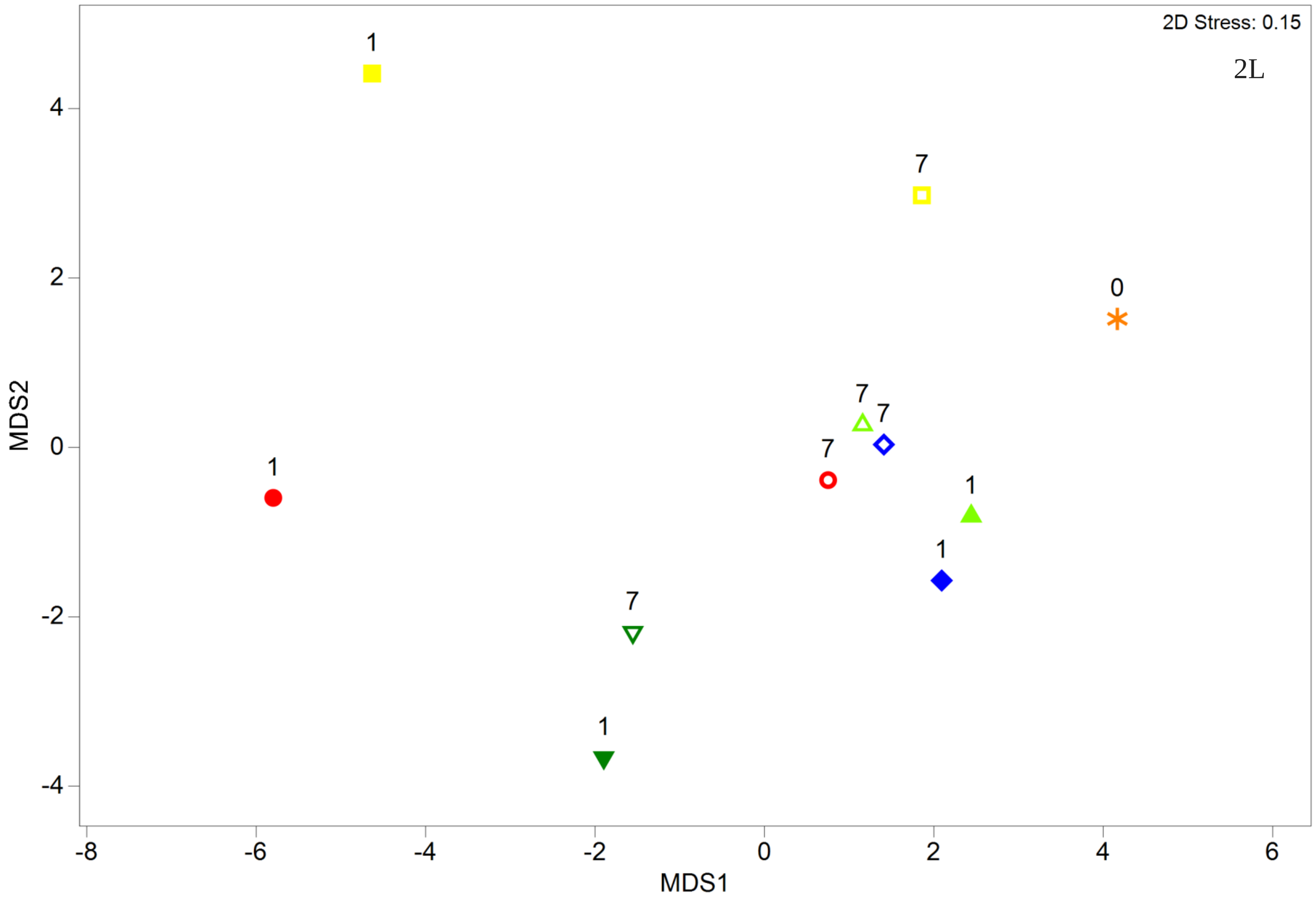
2D Stress: 0.15
2L
MDS2
MDS1
1
7
0
7
7
7
1
1
1
7
1
4
2
0
-2
-4
-8
-6
-4
-2
0
2
4
6

Fig. S2. Metric multi-dimensional scaling plots (mMDS) with an unrestricted zero intercept comparing the distance among the centroids of bacterial population, observed by sequencing 16s rDNA, after being treated with either metallic copper at 2 and 200 µg/ml from core-shell silica copper (CS 2 (light-green triangle) and CS 200 (dark-green triangle) respectively), copper-mancozeb (yellow square), *Ralstonia solanacearum* (red circle), or with water (blue diamond) and a non-treated control (orange star). Soil samples were taken at 0 (orange star), 1 (open shape), or 7 (filled in shape) days post treatment. Datasets were transformed to the 4th root unless otherwise noted. The number represents the trial (1, 2) and the letter represents the resemblance measure used: A = Bray Curtis with a square root transformation, B = Euclidean, C = Chi square, D = Gamma, E = Theta, F = UniFracU, G = UniFracW, H = Gower, I = Gower without joint absences, J = ModGower, K = Sorenson, and L = Jaccard.
